# Dendritic core-shell nickel-iron-copper metal/metal oxide electrode for efficient electrocatalytic water oxidation

**DOI:** 10.1038/s41467-017-02429-9

**Published:** 2018-01-26

**Authors:** Peili Zhang, Lin Li, Dennis Nordlund, Hong Chen, Lizhou Fan, Biaobiao Zhang, Xia Sheng, Quentin Daniel, Licheng Sun

**Affiliations:** 1 Department of Chemistry, KTH Royal Institute of Technology, 10044 Stockholm, Sweden; 2 PULSE Institute, SLAC National Accelerator Laboratory, Stanford University, Menlo Park, CA 94025 USA; 3 Stanford Synchrotron Radiation Lightsource, SLAC National Accelerator Laboratory, Menlo Park, CA 94025 USA; 4 State Key Laboratory of Fine Chemicals, Institute of Artificial Photosynthesis, DUT-KTH Joint Education and Research Centre on Molecular Devices, Dalian University of Technology, 116024 Dalian, China

## Abstract

Electrochemical water splitting requires efficient water oxidation catalysts to accelerate the sluggish kinetics of water oxidation reaction. Here, we report a promisingly dendritic core-shell nickel-iron-copper metal/metal oxide electrode, prepared via dealloying with an electrodeposited nickel-iron-copper alloy as a precursor, as the catalyst for water oxidation. The as-prepared core-shell nickel-iron-copper electrode is characterized with porous oxide shells and metallic cores. This tri-metal-based core-shell nickel-iron-copper electrode exhibits a remarkable activity toward water oxidation in alkaline medium with an overpotential of only 180 mV at a current density of 10 mA cm^−2^. The core-shell NiFeCu electrode exhibits pH-dependent oxygen evolution reaction activity on the reversible hydrogen electrode scale, suggesting that non-concerted proton-electron transfers participate in catalyzing the oxygen evolution reaction. To the best of our knowledge, the as-fabricated core-shell nickel-iron-copper is one of the most promising oxygen evolution catalysts.

## Abstract (web summary)

Splitting water into high-energy fuel represents a renewable way to generate energy, yet the sluggish oxidation kinetics drives up technological costs. Here, the authors prepare tri-metallic core-shell electrodes using nickel, iron, and copper metals to accelerate electricity-driven water splitting.

## Introduction

Water splitting is a sustainable technology to produce hydrogen, which is a carbon-free fuel that offers an attractive route to chemical storage of renewable energy^1^. In practice, water splitting devices need two electrodes: the anode and cathode supported with their respective oxygen evolution reaction (OER) and hydrogen evolution reaction (HER) catalysts. An important property of the catalysts is to reduce the overpotential to drive the half reactions including OER and HER. Among the two half reactions, OER is kinetically sluggish and demands a highly efficient catalyst^2^. Over the past decades, tremendous efforts have been made to synthesize highly efficient, robust, and cost-effective OER catalysts, which has proven difficult. Transition-metal oxides, (oxy)hydroxides, and their derivatives are promising materials thanks to their tunability, abundance, low cost, and potential stability^3, 4^. Up to now, most of the efficient transition metal-based catalysts require overpotentials (*η*) >250 mV to deliver a catalytic current density (*j*) of 10 mA cm^−2^ ^3^. Recently, transition metal phosphides, sulfides, selenides, and alloys have been reported as non-precious OER catalysts with high activity^5–11^. However, further research shows that these materials are precursors that will undergo superficial (Ni_2_P^6^, CoP^7, 8^ NiFe^9^, and Ni_60_Fe_30_Mn_10_ alloy-based electrodes^10^) or complete (Ni, Co, and Fe sulfides^11^ and selenides^12^) transformation into metal oxides and/or (oxy)hydroxides during the catalytic process, revealing that the oxide species formed in situ were responsible for the catalytic activity. In comparison with single-centered metal oxides, bi-, and tri-metal-based catalysts show significantly higher catalytic performance toward OER. The improvement can be attributed to the synergy among the different elements^13, 14^. For instance, NiFe-^15^, NiV-^16^, Ni_60_Fe_30_Mn_10_-^10^, NiFeCr-based^14^ metal oxides, and gelled FeCoW oxyhydroxide^17^ showed better performance than single metal center Ni oxides. These reports inspired us to synthesize tri-transition metal-based alloy as templated precursors to highly active core-shell (CS) metal/metal oxide OER catalysts.

Water oxidation requires four electrons. A catalyst containing multiple redox-active metal ions can buffer the multi-electron transfer processes necessary for OER^14^. Alloying provides a means for mixing a wide range of elements and studying the synergistic effects, but the nature of the physical mixture leads to large crystal size with limited electrochemically accessible surface area and a lack of chemical interaction between different elements^18^. Newly developed fabrication approaches for synthesizing nanostructured alloys by electrodeposition provide a chance to overcome the drawbacks of physical mixture alloys^19^. Hence, nanostructured alloys with multiple redox-active metals may provide new insights into the synergy among different elements and further improve the OER catalytic activity.

Herein, we report a highly active CS metal/metal oxide electrode based on Ni, Fe, and Cu. The parent alloy with dendritic structure is prepared via electrodeposition. This alloy is used as a precursor to fabricate a CS metal/metal oxide electrode with a high electrochemically active surface area (ECSA). This CS–NiFeCu is employed as an electrode for OER, showing an overpotential of only 180 mV for a current density of 10 mA cm^−2^. This is, to the best of our knowledge, the most-efficient OER catalyst in basic media in terms of the overpotential required at 10 mA cm^−2^.

## Results

### Synthesis and characterization of the NiFeCu parent alloy

First, the parent alloy was prepared by electrodeposition, which is one of the most commonly used electrochemical synthesis methods for alloys. This method allows us to control the desired properties, such as the phase composition and the morphology by adjusting the electrochemically deposited conditions^20, 21^. Here, we design the alloy by choosing Ni, Fe, and Cu as the co-depositing elements for the following reasons: (i) single metal center Ni, Fe, and Cu-based oxides have been reported as efficient catalysts for OER;^9, 10, 22^ (ii) The co-depositing species stick easily on the same type but hardly stick to particles of the other type^20^. (iii) Cu species is selected to be corroded to enhance the interfacial surface area^23–25^.

**Fig. 1:**
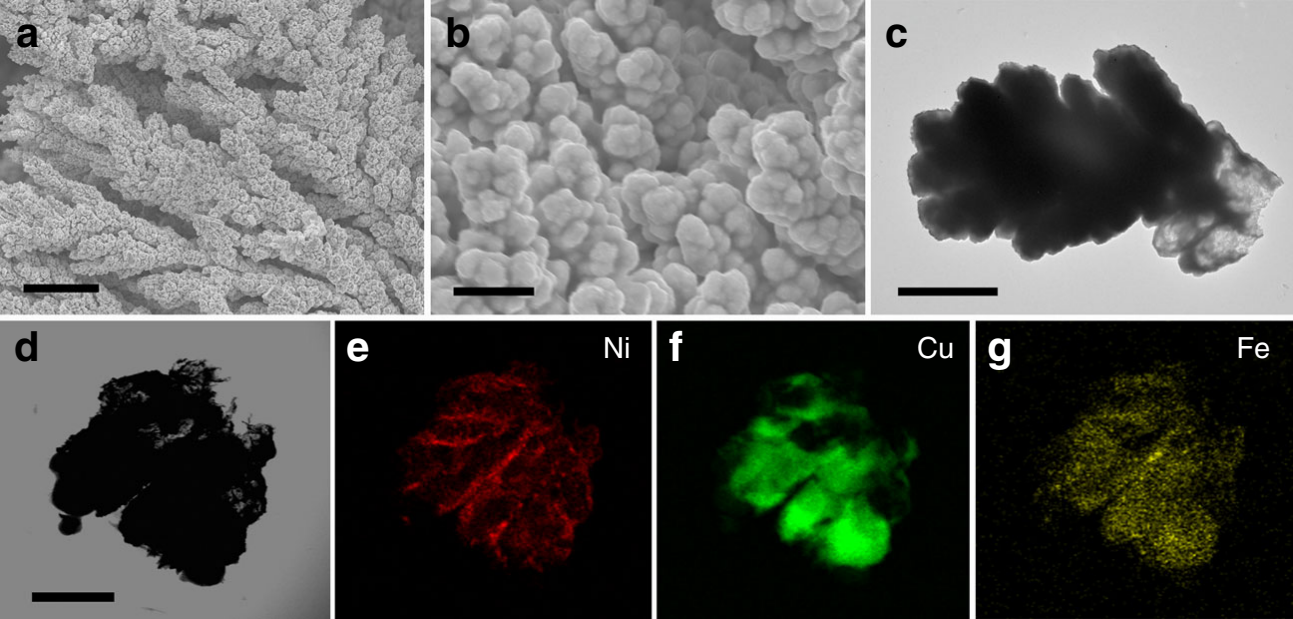
Microscopy measurements of the NiFeCu parent alloy. **a**, **b** SEM images of NiFeCu alloy on nickel foam. Scale bar in **a** is 10 µm, in **b** is 2 µm. **c** TEM image of NiFeCu tip. Scale bar in **c** is 500 nm. **d**–**g** TEM images of a branch tip and corresponding elemental mappings. Scale bar in **d** is 100 nm

The as-designed alloy was prepared by controlled potential electrodeposition on a nickel foam (NF) substrate at −1.10 V vs Ag/AgCl in citrate aqueous solutions containing Ni(Cl)_2_·6H_2_O (80 mM), Fe(NO_3_)_3_·9H_2_O (25 mM) and Cu(SO_4_)·5H_2_O (40 mM) (more details of electrodeposition mechanism, see Supplementary Fig. 1 and Supplementary Note 1). The electrodeposition leads to a brown film deposited on the surface of nickel foam (Supplementary Fig. 1b). This film was characterized by a combination of various techniques. The X-ray powder diffraction (XRD) spectrum of NiFeCu alloy reveals that the as-prepared film formed a solid solution with a face-centered cubic (fcc) structure (Supplementary Fig. 2)^20^. The surface of the NiFeCu alloy was studied by scanning electron microscopy (SEM) and transmission electron microscopy (TEM). SEM images observe that NiFeCu alloy uniformly deposited onto the nickel foam substrate with a dendritic structure (Fig. 1a) with clearly distinguishable hierarchy and tip-splitting, as shown in Fig. 1a–c. The diameter of branches is distributed in 1.5–3.0 μm. Elemental mapping analysis of TEM (Fig. 1d–g) showed that Fe is homogeneously distributed in the film. Copper prevails at the convex parts, whereas nickel is distributed in the concave ones^20^. Further analysis of the as-deposited ternary system by energy dispersive X-ray spectroscopy (EDS) showed that Ni, Fe, Cu are the principal elemental components with an atomic ratio of Ni:Fe:Cu = 10:1:21 (Supplementary Fig. 3).

### Synthesis and characterization of the CS-NiFeCu electrode

Next, we produced the metal/metal oxide CS–NiFeCu electrode by dealloying. Dealloying is a commonly used top-down nano-synthesis technique where one or more chemically active elements are selectively oxidized and removed from parent alloy by chemical and/or electrochemical methods^10, 26–28^. This keeps the metallic core at high electrical conductivity, which enhances the facile electron transfer during catalytic process. The metal oxide shell is formed on top of the metallic core, which makes the oxide the active catalyst for OER. In details, the CS-NiFeCu electrode was obtained from the NiFeCu parent alloy by dealloying via potentiostat control using a positive bias voltage at the current density of 200 mA cm^−2^ in an alkaline solution for 10 h (Supplementary Fig. 4). Under these dealloying conditions, copper shows the highest chemical activity relative to nickel and iron. Therefore, during the process, loaded Cu on the surface was gradually dissolved into the solution, whereas a metal oxide layer was generated on the surface, preventing the metallic core from further corrosion (Supplementary Fig. 1c and Supplementary Note 2)^29^. We note that a complete oxidized structure is not desirable owing to the poor electrical conductivity of most metal oxides^10, 30^.

**Fig. 2:**
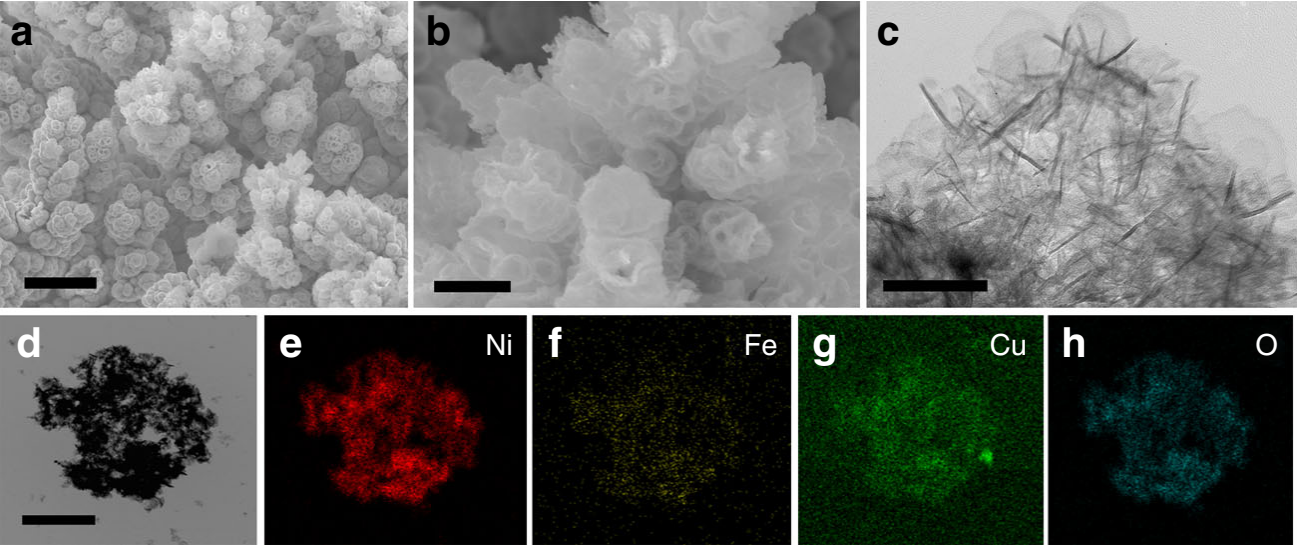
Microscopy measurements of the CS-NiFeCu electrode. **a**, **b** SEM images of CS-NiFeCu catalyst on nickel foam. Scale bar in** a** is 10 µm, in** b** is 2 µm. **c** TEM image of CS-NiFeCu shell. Scale bar in **c** is 50 nm. **d**–**h** TEM images of CS-NiFeCu shell and corresponding elemental mappings. Scale bar in **d** is 100 nm

The surface of the CS-NiFeCu was studied by SEM and TEM. SEM images show that the CS-NiFeCu electrode had a similar morphology compared with its parent alloy (Fig. 2a). Dendritic structure with moss-like branches was observed by SEM images (Fig. 2a, b). The diameter of branches is distributed in 1.5–3.0 μm range, similar to its parent alloy. Further zoom in SEM images observed that the Cu prevails in the convex parts of the surface were eroded, leaving a porous shell (Fig. 2b). Magnified TEM image shows that the CS-NiFeCu oxides shell cover the branches with a thickness of 250–300 nm (Fig. 2c and Supplementary Fig. 5).

The XRD patterns (Supplementary Fig. 2) for the CS-NiFeCu catalyst show a series of peaks at 2*θ* = 43.4°, 50.4°, 74.2°, 90.0°, and 95.3°, at the same positions as the fcc NiFeCu parent alloy^20^. Two weak diffraction peaks of CuO at 2*θ* = 35.6° and 38.9° were observed, corresponding to the residual edged copper^31^. The silence of new diffraction signals in the detection window confirms that the NiFe oxides and/or hydroxides shell surrounding the metallic core in CS-NiFeCu electrode are amorphous in nature^9, 32^. The XRD analysis of the bulk CS-NiFeCu indicated that only a small amount of the parent alloy was transformed into oxide during dealloying.

To better understand the elemental composition and degree of oxidation, EDS analysis was applied to the bulk material and the oxide shell of CS-NiFeCu. EDS analysis of the bulk CS-NiFeCu catalyst shown an elemental composition (atomic) of Ni, Fe, Cu, O at Ni:Fe:Cu:O = 10:1:16:3 (Supplementary Fig. 6) compared with Ni:Fe:Cu = 10:1:21 for the parent alloy NiFeCu. The metal to oxygen ratio in the CS-NiFeCu catalyst is 37:3, which is far from full oxidation. The reduced amount of Cu present in CS-NiFeCu suggests the loss of Cu during the dealloying process. During TEM analysis, oxide shell debris was selected for further EDS analysis (Supplementary Fig. 7), showing an atomic proportion of Ni:Fe:Cu:O = 10:1:10:26, which is consistent with an oxide surface of CS-NiFeCu.

**Fig. 3:**
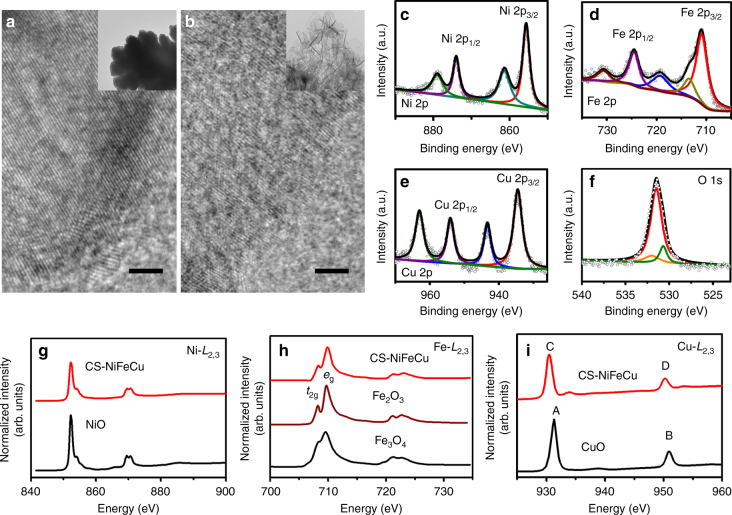
HRTEM, XPS, and XAS measurements. High-resolution TEM images of NiFeCu alloy (**a**) and CS-NiFeCu (**b**), and the insets are the low magnification view for the overall materials. Scale bars in **a** and **b** are 2 nm. XPS spectra of Ni 2p (**c**), Fe 2p (**d**), Cu 2p (**e**) and O 1s (**f**) on the surface of CS-NiFeCu electrode. Normalized soft-XAS Ni *L*_2,3_-edge spectra (**g**), Fe *L*_2,3_-edge spectra (**h**) and Cu *L*_2,3_-edge spectra (**i**) of CS-NiFeCu electrode together with NiO, Fe_3_O_4_, Fe_2_O_3_, and CuO as references

Elemental mapping analysis of the oxide shell (Fig. 2d–h) show that Ni, Cu, Fe, and O are homogeneously distributed in the oxide shell, and the initial Cu-rich parent alloy taps have lost about half of its Cu during the dealloying process. High-resolution TEM image of CS-NiFeCu oxide shell show lattice fringes typical of an amorphous (disorder) material, which is different from that of the crystalline parent alloy (Fig. 3a, b). The selected area electron diffraction (SAED) pattern of the shell is typically a halo feature, further indicated that the oxide shell is in the amorphous state (Supplementary Fig. 8). The SEM, TEM, EDS, and XRD analysis of the bulk CS-NiFeCu clearly indicated that only the surface of the parent alloy was transformed into oxide shell. Furthermore, a loading amount of 10.2 ± 0.5 mg cm^−2^ was detected by weight (High precision electronic balances, Mettler Toledo). Among them, ~1.6 mg cm^−2^ of that was contributed to OER, roughly calculated by the accumulated charge for Ni^2+^/Ni^3+^ transformation (more details, see Supplementary Fig. 9 and Supplementary Note 3)^9^.

The electronic states of the CS-NiFeCu electrode surface were further analyzed by X-ray photoelectron spectroscopy (XPS). In the XPS spectra, Ni shows a distinct 2p_3/2_ peak around 855.7 eV (Fig. 3c) along with a strong satellite indicative of divalent Ni. Iron shows a fairly broad Fe 2p_3/2_ peak around 710.8 eV along with a small but significant satellite near 720 eV that is associated with Fe^3+^ (Fig. 3d). The main Cu 2p_3/2_ peak (934.1 eV) is accompanied with a strong satellite that can be uniquely ascribed to divalent Cu (Fig. 3e). The XPS spectra are thus consistent with an oxidized surface consisting primarily of Ni^2+^, Fe^3+^, and Cu^2+^ oxide^15, 31^. The main peak in the O 1s spectrum was at 530.6 eV (Fig. 3f), attributed to metal oxide^15, 31^.

To better understand the valence state of Ni, Fe, and Cu in these samples, we have measured X-ray absorption spectroscopy (XAS) on the Ni, Fe, and Cu *L*_2,3_-edges. Metal *L*-edge XAS is a powerful probe of the local electronic structure that displays sensitive to the valency, spin, and symmetry of the metal atom. Figure 3g shows the normalized soft-XAS Ni *L*_2,3_-edge spectra of CS-NiFeCu in comparison with NiO as a reference. The CS-NiFeCu is nearly identical to NiO apart from a slight broadening, which suggests that the oxidized Ni at the surface is primarily divalent Ni^2+^ ^33^. Figure 3h shows the Fe *L*_2,3_ spectra of CS-NiFeCu together with powder references from Fe_2_O_3_ (Fe^3+^) and Fe_3_O_4_ (two types of Fe^3+^ and one 3rd Fe^2+^). The CS-NiFeCu is very similar to Fe_2_O_3_, showing the characteristic absorption profile of high spin Fe^3+^ in octahedral coordination^34^. Figure 3i shows normalized soft-XAS Cu *L*_2,3_-edge spectra of CS-NiFeCu in comparison with CuO as a reference. The energy separation between *L*_3_ (Cu 2p_3/2_) and *L*_2_ (2p_1/2_) is determined by the spin-orbit coupling, and it is dependent on the oxidation states of Cu, which is 19.0 eV for CuO and 21.0 eV for Cu_2_O^35^. Our measurements show the *L*_3_-*L*_2_ splitting is 19.7 eV for CuO, which is similar to the *L*_3_-*L*_2_ splitting in CS-NiFeCu. We note that there is 0.7 eV shift to lower energy compared with peaks in CuO. This shift could be related to the different configuration of Cu atoms and more electron density in Cu atoms in CS-NiFeCu compared with CuO^36^. The small peak shifted by 2.1 eV from the main peak is attributed to a metallic copper peak^36^. These data were collected in total electron yield (TEY). In addition to it, we also measured fluorescence yield (FY) simultaneously shown in supporting information (Supplementary Fig. 10). The FY probe deeper than TEY measurements. In fact, they show the expected results that more of the metallic core can be observed in FY measurements when the measurements probe deeper.

It is well established that the X-ray absorption spectra at O *K*-edge probe give rise to a significant pre-edge intensity in transition-metal complexes owing to the hybridization of the O 2p states with the TM 3d state that become accessible through the O *K*-edge dipole transitions (1s->2p)^37^. Supplementary Fig. 11 shows the normalized XAS O *K*-edge spectra of CS-NiFeCu in comparison with CuO, FeO, Fe_2_O_3_, and NiO references. CS-NiFeCu shows numerous peaks buried in a broad feature representative of the mixture of hybridized O 2p-TM 3d states. It is outside of the scope of this article to disentangle these contributions.

### Oxygen-evolution catalysis

**Fig. 4:**
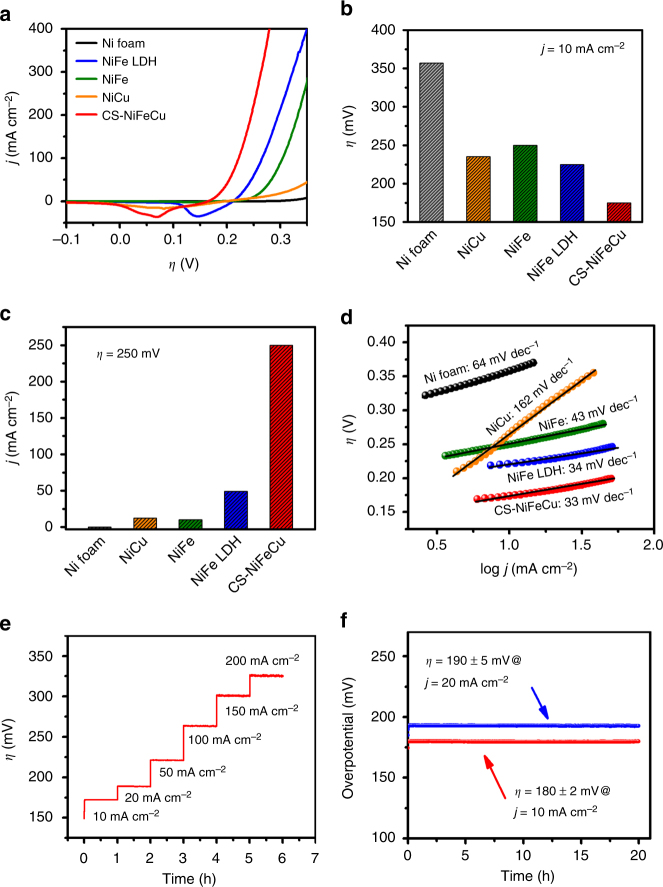
Electrochemical characterizations for OER. **a** Polarization curves of CS-NiFeCu, NiFe LDH, NiFe, NiCu electrodes, and nickel foam. **b** The overpotentials required for* j *= 10 mA cm^−2^ with different electrodes. **c** Current densities at *η* = 250 mV with different electrodes. **d** Tafel plots for nickel foam, NiCu, NiFe LDH, NiFe, and CS-NiFeCu electrodes. **e** Chronopotentiometric measurements of OER in 1.0 M KOH at various current densities using CS-NiFeCu as a catalyst. **f** Extended chronopotentiometric measurements at *j *= 10 mA cm^−2^ and* j *= 20 mA cm^−2^ for 20 h

The electrocatalytic performances of CS-NiFeCu, NiFe, NiCu, and Ni foam toward OER in 1 M KOH aqueous solution were measured as shown in Fig. 4. NiFe and NiCu electrodes were prepared under the same conditions as CS-NiFeCu, but without the participation of the third metal Cu or Fe, respectively (Supplementary Fig. 12). The state-of-the-art NiFe LDH (layered double hydroxide) catalyst was prepared as a previously reported method for direct comparison^38, 39^. All the data were recorded in a standard three-electrode electrochemical cell using an Ag/AgCl (3 M KCl) as a reference electrode and a Pt plate (4 cm^2^) as the counter electrode. The polarization curve of CS-NiFeCu shows the best catalytic activity among the five electrodes, delivering much higher current density at the same overpotential than the others (Fig. 4a). For further clear comparison of the analysis data, the overpotentials at a catalytic current density of 10 mA cm^−2^ were plotted and shown in Fig. 4b. The CS-NiFeCu electrode required an overpotential of 180 mV, which was 66, 79, 81, and 182 mV lower than that of NiFe LDH, NiFe, NiCu, and nickel foam, respectively (Fig. 4b). Another way to view the data is to fix the overpotential at *η* *=* 250 mV, as shown in Fig. 4c. The CS-NiFeCu electrode delivered a current density of 248 mA cm^−2^, which was 8.5, 32.6, 26.8, and 3846-fold higher than those of NiFe LDH, NiFe, NiCu, and nickel foam, respectively. As a matter of fact, the catalytic performance of CS-NiFeCu in 1 M KOH solution is superior to the previously reported catalysts including NiFe LDH (Supplementary Table 1). So far, NiFe LDH has been generally regarded as the most active OER catalysts in alkaline conditions^38^. To further enhance the activity, the advanced hydrothermal synthesis and electrosynthesis methods have been applied^32, 39^. However, an overpotential of 224 mV for *j* = 10 mA cm^−2^ was still required, even for the best method^39^. The activity of NiFe LDH was further improved by hybrid approaches with carbon nanomaterials. For example, NiFe hydroxide/graphene superlattice^40^, exfoliated graphene/Co_0.85_Se/NiFe LDH composites^41^, and r-GO/NiFe LDH^42^ (r-GO, reduced graphite oxide) reached *j* = 10 mA cm^−2^ at the overpotentials of 210, 203, and 195 mV, respectively. Recently, selenide-derived NiFe oxide was reported by Hu et al., which delivers a current density of 10 mA cm^−2^ at an overpotential of 195 mV^12^. In short, our as-prepared CS-NiFeCu is now the most-efficient OER catalyst in basic media in terms of the overpotential that required at 10 mA cm^−2^.

The kinetic parameters of the five electrodes were obtained from the corresponding polarization curves by plotting overpotential against log (*j*) (Fig. 4d). The as-prepared CS-NiFeCu electrode shows a Tafel slope of 33 mV dec^−1^, smaller than those of other catalysts (34−162 mV dec^−1^).

What is noteworthy is that the bimetal-based electrodes show lower activity than CS-NiFeCu (Fig. 4a–d). These results indicate that the outstanding activity of CS-NiFeCu is based on the three indispensable elementals rather than any two of them. The preparation of CuFe under the same electrodeposited conditions, but without the participation of Ni, was not successful (Supplementary Fig. 13 and Supplementary Note 4), indicating that Ni promoted the co-electrodeposition of Fe and Cu (more details of electrodeposition mechanism, see Supplementary Fig. 1 and Supplementary Note 1).

In order to test the steady-state activity and durability of CS-NiFeCu, multiple current steps of chronopotentiometry experiments were conducted in 1 M KOH. As shown in Fig. 4e, the corresponding change of applied potentials was profiled when the catalytic current density was increased from 10 to 200 mA cm^−2^. Beginning with 10 mA cm^−2^, the overpotential rapidly levels off at 180 mV and remains constant for the following 1 h. Further increase the multiple current steps leads to the overpotentials persisted constantly at a higher value than that at 10 mA cm^−2^. It is noticed that the rough line shown for higher currents than 50 mA cm^−2^ is due to the bubble formation during water oxidation reactions. Furthermore, the durability of the CS-NiFeCu anode during water oxidation was tested at a constant current density of 10 and 20 mA cm^−2^ for 20 h. As shown in Fig. 4f, the overpotential remained at 180 ± 2 mV during 20 h to keep a current density of 10 mA cm^−2^. The same behavior was observed at 20 mA cm^−2^ with an applied overpotential of 190 ± 5 mV. After 20 hours electrolysis, the amount of generated oxygen gas was measured in addition to the recorded current and charge, which were used for the calculation of the Faradaic efficiency^10, 16^. The Faradaic efficiencies of 93% and 98% were obtained at *j* = 10 and 20 mA cm^−2^, respectively (Supplementary Fig. 14), indicating that the accumulated charge was almost consumed on water oxidation. These responses demonstrate the excellent catalytic activity, mass transport properties, and intrinsic robustness of the CS-NiFeCu electrode for prolonged electrolysis upon water oxidation.

## Discussion

**Fig. 5:**
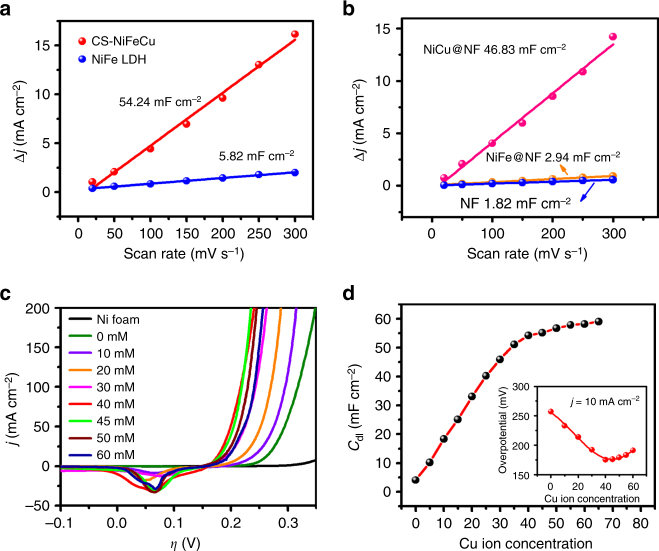
Contributing factors to the electrochemically active surface area. **a** Capacitive *j* vs scan rate for CS-NiFeCu and NiFe LDH anodes. **b** Capacitive *j* vs scan rate for NiCu and NiFe and Ni anodes. For **a** and **b**, the linear slope is equivalent to twice of the double-layer capacitance *C*_dl_. **c** Polarization curves of CS-NiFeCux electrodes. CS-NiFeCux electrodes are prepared in same method as CS-NiFeCu with constant concentration of Ni^2+^ (80 mM) and Fe^3+^ (25 mM), and various concentrations of Cu^2+^ (0–60 mM) in the electrodeposited electrolytes. **d** *C*_dl_ of CS-NiFeCux anodes. Inset, overpotential required for *j* = 10 mA cm^−2^

ECSA is an influential factor for OER catalysts. An increase of ECSA normally leads to the enlargement of the active sites and results in the enhancement of catalytic performance^10, 12, 16^. Herein, we compare the ECSA of each electrode, which was obtained from cyclic voltammetry (CV) curves for direct comparison (Fig. 5a). In details, by plotting the Δ*j* (|*j*_charge_−*j*_off charge_|) at Faradaic silence potential range against the scan rates, the linear slope is obtained, which is a positive correlation with the double-layer capacitance (*C*_dl_), and been used to represent the corresponding ECSA^12, 16^. It is important to note that CS-NiFeCu has dramatically higher ECSA comparing with NiFe LDH. As shown in Fig. 5a, the linear slope (*C*_dl_) of CS-NiFeCu electrode has a value of 54.24 mF cm^−2^, 9.3-fold higher than that of NiFe LDH under the same conditions. The significantly high ECSA of CS-NiFeCu might be attributed to the porous oxide shells, which were created during dealloying process.

In order to distinguish the structural contribution of each element, the double-layer capacitance (*C*_dl_) of NiFe and NiCu electrodes were collected. NiCu shows the *C*_dl_ value of 46.83 mF cm^−2^ close to CS-NiFeCu, which is ~16-fold of NiFe and 26-fold of Ni foam (Fig. 5b). This evidence indicated that the high ECSA of CS-NiFeCu was mainly based on NiCu rather than NiFe. Further detail effect of Cu toward the ECSA in CS-NiFeCu_x_ was detected by tuning its content variation in the film (Fig. 5c, d). As shown in Fig. 5d, the *C*_dl_ of CS-NiFeCu_x_ was enlarged with the increase of Cu ion concentration in the electrodeposited electrolytes. The best catalyst for OER was obtained at the concentration of 40 mM (Cu^2+^) (Fig. 5c and Supplementary Fig. 15), which leads to the formation of an alloy with an atomic ratio of Ni:Fe:Cu = 10:1:21. Further enhancing the content of Cu in the electrodeposited film by increasing the concentration of Cu^2+^ in the electrodeposited electrolyte lead to a mild decrease in activity, which may result from the excessively loose and friable structure.

In order to investigate the intrinsic activity of each electrode, the polarization curves of CS-NiFeCu, NiFe LDH, NiFe, NiCu, and Ni foam are normalized to ECSA, respectively (Supplementary Fig. 16). The normalized CS-NiFeCu curve achieves a 1 mA cm^−2^ current density at *η* = 185 mV, while NiFe LDH, NiFe, NiCu, and Ni foam require the overpotentials of 228, 260, 300, and 320 mV to reach the same current density. The overpotential (@ *j*_normalized_ = 1 mA cm^−2^) of CS-NiFeCu for OER was cathodically shift by 43 mV compared with NiFe LDH and 75 mV compared with NiFe (Fig. 5c). These results indicated that CS-NiFeCu has the optimal intrinsic activity compared with other four electrodes.

So far, two fundamentally different mechanisms were reported to understand how materials catalyze the OER^43^. Based on the relationship between pH values and OER activity, the OER mechanisms were divided into two types. First, the four concerted proton-electron transfer pathway with pH-independent activity on the reversible hydrogen electrode (RHE) scale, where the catalytic performance was contributed to the surface metal-ion centers^44^. Second, the four non-concerted proton-electron transfer mechanism with pH-dependent activity on the RHE scale, where the catalytic performance was contributed to the bulk material, including the surface metal-ion centers and the lattice oxygen^43^.

**Fig. 6:**
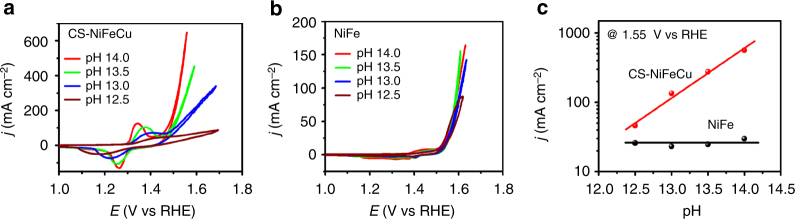
pH-dependent OER activity on the RHE scale. **a**, **b** Cyclic voltammetry measurements from O_2_-saturated 0.032 M KOH (pH 12.5) to 1 M KOH (pH 14) recorded at 10 mV s^−1^ with CS-NiFeCu and NiFe electrodes. **c** Specific OER activity (current density) at 1.55 V vs RHE after *iR* correction as a function of pH

CV measurements recorded from 0.032 M (pH 12.5) to 1 M KOH were used to investigate the relationship between pH values and OER activity (Fig. 6), that provide valuable information for studying the catalytic mechanism^43^. RHE scale was used as the reference to assure that the OER overpotential remained identical across different values of pH with respect to the equilibrium O_2_/H_2_O redox potential^45^. The CV of CS-NiFeCu shows an oxidation peak current density as large as 126 mA cm^−2^ at 1.35 V, corresponding to the Ni^2+^/Ni^3+^ oxidation in 1 M KOH, which is ~10 times as that of NiFe LDH supported with nickel foam^32^. This extremely high peak current density is attributed to the large ECSA of CS-NiFeCu, which is 9.3-fold higher than that of NiFe LDH. As shown in Fig. 6a, the OER activity of CS-NiFeCu at 1.55 V vs RHE exhibits pH-dependent OER activity on RHE scale (Fig. 6a, c), hinting that non-concerted proton-electron transfers engage in catalyzing the OER^46^, in which the rate-limiting step is either a proton transfer step or preceded by acid/base equilibrium. With this mechanism, the high activity of CS-NiFeCu can be contributed to bulk oxide shell, including the surface metal-ion centers and the lattice oxygen^43^. Without the participation of copper, dimetal-based NiFe electrode show pH-independent of OER activity on RHE scale (Fig. 6b, c). These results indicate that the NiFe electrode catalyzed the OER with concerted proton-electron transfer steps, where the activity is determined by the reaction intermediate binding strengths on surfaces^44^. Above research results indicated that the participation of Cu into NiFe led to a fundamentally different mechanism for the OER. In addition to high ECSA factor, it is the bulk material-based mechanism, which was triggered by the participation of Cu, leads to the OER activity enhancement on CS-NiFeCu electrode.

In order to get more subtle electrochemical signals, nickel microelectrodes with a surface area of 0.0078 cm^2^ (*d* = 0.1 cm) were used as the substrates. The CVs for Ni and NiFe electrodes in KOH exhibit two primary features, a redox couple at 1.38 V and 1.41 V vs RHE and an oxidation current visible at overpotentials greater than 1.45 V vs RHE (Supplementary Fig. 17a). The redox peaks are attributed to the transformation between Ni^2+^ and Ni^3+^ ^47, 48^. Oxidation currents at higher potentials are due to oxygen evolution. CVs for CS-NiFeCu_x_ series differ noticeably in their Ni^2+^/Ni^3+^ redox characteristics (Supplementary Fig. 17b). As more Cu is incorporated into the NiFe-based film, the Ni^2+^/Ni^3+^ redox peaks were cathodically shifted to 1.33 V vs RHE, a new Ni^2+^/Ni^3+^ oxidation peak was generated at 1.35 V vs RHE, which further enhanced with the increase of Cu amount in the electrodeposited electrolyte (Supplementary Fig. 17b). The Ni^2+^/Ni^3+^ oxidation peak of the as-prepared CS-NiFeCu shifted to a negative potential by as much as 50 mV relative to that for NiFe. This result suggests that the electrochemical oxidation of Ni^2+^ to Ni^3+^ is facilitated by the presence of Cu. The CV for CS-NiFeCu shows two adjacent Ni^2+^/ Ni^3+^ oxidation peaks, indicated that two kinds of electroactive components exist in the oxide shell of CS-NiFeCu. The peak at 1.35 V vs RHE is corresponding to NiFeCu oxide and the peak at 1.38 V vs RHE is corresponding to NiFe oxide. Both NiFeCu oxide and NiFe oxide are active for OER. NiFeCu oxide, characterized with a more negative Ni^2+^/Ni^3+^ oxidation peak, has the pivotal role to reduce the overpotential for OER.

**Fig. 7:**
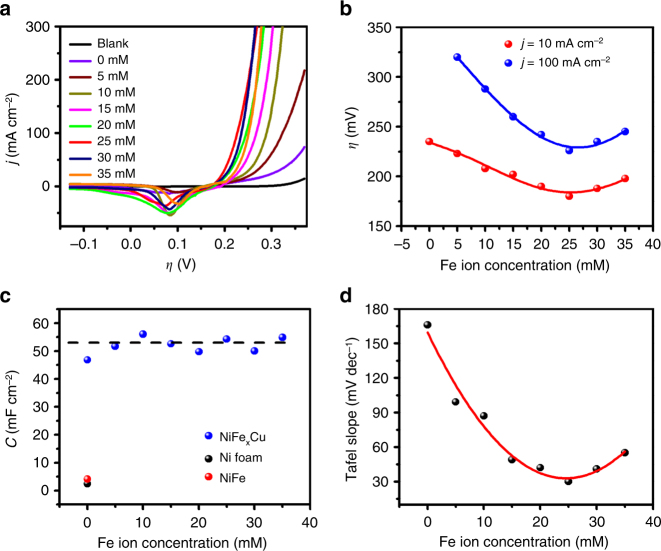
Contributing factors to the kinetics. **a** Polarization curves of CS-NiFexCu anodes. **b** Overpotentials required for *j* = 10 and 100 mA cm^−2^ with CS-NiFe_x_Cu anodes in 1 M KOH. **c** *C*_dl_ of CS-NiFexCu anodes. **d** Tafel slopes of CS-NiFe_x_Cu in 1 M KOH. CS-NiFe_x_Cu electrodes are prepared in the electrodeposited electrolytes with concentration of Ni^2+^ (80 mM), Cu^2+^ (40 mM), and variable concentration of Fe^3+^ (0−35 mM) concentrations

As well known, the doping of Fe in Ni-based catalysts can enhance the catalytic activity^42, 49^. To verify the role of Fe in CS-NiFeCu, electrodes with a constant concentration of NiCu and a variable concentration of Fe were prepared by adjusting the Fe ion concentration in the electrodeposited electrolytes. With the increase of Fe ion concentration, polarization curves of CS-NiFe_x_Cu show obviously enhancement in catalytic performance (Fig. 7a, b). The current densities at *η* = 250 mV were increased from ~20–200 mA cm^−2^. As shown in Fig. 7a, the activity of CS-NiFe_x_Cu increases with Fe content, further confirmed that the NiFe-based components are the active sites for water oxidation reaction^9^. ECSAs of CS-NiFe_x_Cu anodes were further examined in details, as shown in Fig. 7c. The *C*_dl_ of CS-NiFe_x_Cu electrodes with different Fe content distributed in the range of 50 ± 5 mF cm^−2^ (Fig. 7c). No obvious change was observed with the increase of Fe content, indicated that the ECSA of CS-NiFe_x_Cu electrodes is independent of Fe. This result ruled out the contribution of Fe to the ECSA factor.

Tafel slope analysis was used to understand the influence of Fe content on the intrinsic kinetics of CS-NiFe_x_Cu. As shown in Fig. 7d and Supplementary Fig. 18, the Tafel slope is crucially affected by Fe content. NiCu electrode without Fe shows a Tafel slope of 164 mV dec^−1^ in 1.0 M KOH. The Tafel slopes for OER reduced to the smallest 33 mV dec^−1^ when the Fe^3+^ concentrations were increased from 0 to 25 mM, while the Fe:Ni atomic ratio in the electrodeposited film was increased from 0 to 10:1. However, further increasing the amount of Fe in CS-NiFe_x_Cu electrodes led to a moderate decrease of Tafel slope values. When the Fe^3+^ concentrations were increased from 25 mM to 35 mM, the Fe:Ni atomic ratio in the electrodeposited film increased from 0.1 to 0.14, and the corresponding Tafel slopes decrease from 34.0 to 44 mV dec^−1^. This result strongly supports the conclusion that doping Fe into NiCu based materials can adjust the catalytic property by tuning the intrinsic kinetics. Furthermore, it is important to notice that there is always an optimal proportion that leads to the best performance. This result is slightly different from the previous studies that the NiFeOx activity varied marginally when the Fe:Ni atomic ratio was adjusted from 0.1 to 0.55^42, 49^. The relationship between the optimal proportion and catalytic activity was still not clear and needs more investigation.

In summary, using electrodeposited NiFeCu as a precursor, we have shown that the tri-metal-based alloy can be converted into the CS metal/metal oxide catalyst CS-NiFeCu for OER. The high catalytic performance of CS-NiFeCu for water oxidation is attributed to the synergistic effect of Ni, Fe, and Cu. The relative amounts of the three elements in CS-NiFeCu electrode play a decisive role by enhancing the ECSA and intrinsic catalytic activity. A current density of 10 mA cm^−2^ is obtained at an overpotential of only 180 mV in 1 M KOH alkaline solution using CS-NiFeCu as working electrode, which is one of the most active OER catalysts reported to date. The nano-synthesis approach described in this work may be applicable to the development of other alloy-based nanomaterials as catalysts for OER and beyond.

## Methods

### Preparation of CS-NiFeCu electrode

The CS-NiFeCu anodes were prepared through two steps: (i) NiFeCu parent alloy was grown on nickel foam (thickness: 1.6 mm, bulk density: 0.45 g cm^−3^, Goodfellow) via electrodeposition; (ii) parent alloys were converted into CS-NiFeCu anode via a dealloying treatment (more details of electrodeposition mechanism, see Supplementary Fig. 1). Before electrodeposition, the nickel foam was sonicated in concentrated HCl (37%) solution for 5 min to remove the NiO_x_ surface layer, and subsequently rinsed with water and acetone, then allowed to dry in air. The electrodeposition was carried out with a standard three-electrode electrochemical cell containing nickel foam as the working electrode, a platinum foil (4 cm^2^) auxiliary electrode and an Ag/AgCl (3 M KCl) reference electrode. The electrolyte solution consisted of 80 mM NiCl_2_·6H_2_O, 0**−**25 mM Fe(NO_3_)_2_·9H_2_O, 0**−**40 mM Cu(SO_4_)·5H_2_O, 0.2 M Sodium citrate tribasic dehydrate (Na_3_C_6_H_5_O_7_·2H_2_O), 0.45 M ammonium sulfate ((NH_4_)_2_SO_4_) and 0.55 M sodium hypophosphite monohydrate (NaPO_2_H_2_·H_2_O). The electrodeposition solution pH values of were adjusted to 10.0 by 10 M NaOH solution. To optimize the deposited NiFeCu alloy compositions, the concentrations of Ni^2+^, Fe^3+^, and Cu^2+^ in electrolyte were tuned to systematically varied the Ni:Fe:Cu atomic ratio in the electrodeposited parent alloy film. We found that an 80:25:40 molar ratio of Ni^2+^, Fe^3+^, and Cu^2+^ provides the CS-NiFeCu electrode with the highest catalytic OER activity. The CS-NiFeCu in this work was prepared with the 80:25:40 molar ratio of Ni^2+^, Fe^3+^, and Cu^2+^. Typically, the NiFeCu alloy film was prepared though controlled potential electrolysis at **−**1.1 V vs Ag/AgCl at room temperature using a CH Instruments 660E potentiostat. The optimized NiFeCu alloy deposition time has been determined to be 1 h. After deposition, the alloy was carefully rinsed with water and acetone. The dealloying step was carried out in 3 M KOH with a controlled current density of 200 mA cm^−2^ for 10 h. After dealloying, the CS-NiFeCu anode was withdrawn carefully from the electrolyte, rinsed with water and acetone, and then allowed to dry in air.

### Physical characterization

XPS characterization was acquired with a Thermo VG ESCALAB250 surface analysis system. Powder XRD measurement was performed on a X’Pert PANalytical Pro MRD. SEM images and EDX spectra were obtained with a NOVA NanoSEM 450 and JEOL JSM 7401 equipped with EDX system. TEM images and SAED were taken on JEOL JEM2100 TEM.

### Soft-XAS

Ni, Fe, and Cu *L*_2,3_-edge and O *K*-edge soft-XAS of samples were performed at beamline 8-2 of the Stanford Synchrotron Radiation Lightsource (SSRL), SLAC National Accelerator Laboratory, USA. All samples were attached to an aluminum sample holder using double-sided conductive carbon tape. The *L*_2,3_-edge spectra were collected at room temperature under ultrahigh vacuum (10−9 Torr) with the incident beam monochromatized using a spherical grating monochromator with 1100 mm^−1^ ruling, where the exit and entrance slits were set to an intermediate resolution of 0.3 eV. All XAS spectra were collected in the TEY method, obtained through the drain current, and corresponding to probing depths of 2–5 nm. The NiO, Fe_3_O_4_, Fe_2_O_3_, and CuO standard powders were applied as ordered directly onto the carbon tape. The spectra were normalized to the incident photon flux measured by a gold grid upstream. The spectra were normalized via a linear background subtraction followed by an intensity normalization (maximum set to 1) for visual comparison.

### Electrochemical characterization

All electrochemical experiments were performed with a CH Instrument 660E potentiostat. The electrochemical cell containing samples as the working electrode, a platinum foil (4 cm^2^) auxiliary electrode and BASi Ag/AgCl reference electrode. In daily experiments, a Hg/HgO (1 M KOH, Tjaida) electrode was used as a standard reference (*E*_Hg/HgO_ = 0.098 V vs RHE). When Ag/AgCl electrode was used as a reference electrode in alkaline aqueous electrolytes, the potential differences (Δ*E*) between Ag/AgCl electrode and Hg/HgO electrode in the same alkaline electrolyte were recorded for calibration (*E*^o^_Hg/HgO_ _=_ *E*^o^_Ag/AgCl_−|Δ*E*| = 0.098 V vs RHE). The reference electrodes were calibrated by measuring the RHE potential using a Pt electrode under H_2_ atmosphere once a week. In 1 M KOH, all potentials measured were converted to the overpotential reference scale using the Nernst equation: *η* = *E*_Ag/AgCl_−0.221 V. Potentials converted to RHE reference scale via the Nernst equation: *E*_RHE_ = *E*_Ag/AgCl_ + 0.059 pH + *E*^o^_Ag/AgCl_. The as-prepared anodes were fixed to 0.5 cm^2^ by using epoxy glue. Platinum foil (4 cm^2^) was purchased from Tjaida and used as counter electrode. The internal resistance between the reference and working electrodes (*R*u) has been measured by automatic current interrupt method and impedance method. In automatic measurements, the value of *R*u was in the range of 0.7–0.9 Ω, which is small and similar to the impedance data (0.75–0.78 Ω) (Supplementary Fig. 19). In this paper, the *iR* compensation was performed by automatic current interrupt method with a value of 75% × *R*u through the CH instrument 660E potentiostat. In order to provide reliable electrochemical data and avoid the overlap between Ni^2+^/Ni^3+^ oxidation and OER, polarization curves were recorded from high initial potentials to low final potentials with a 5 mV s^–1^ scan rate (more details, see Supplementary Fig. 20 and Supplementary Note 5). Before sweep, the as-prepared anodes were activated by a chronopotentiometry scan with the 50 mA cm^−2^ current density for 2 h. Tafel slopes were calculated using the Polarization curves by plotting overpotential against log(current density). The steady-state activity and long-term activity were evaluated by chronopotentiometry measurements. The ECSA was determined by measuring the capacitive current associated with double-layer charging from the scan rate CV-dependence. Here, the CV potential window was 0.3 to –0.5 vs Ag/AgCl. The scan rates were 20, 50, 100, 250, 200, 250, and 300 mV s^–1^. The double-layer capacitance (*C*_dl_) was estimated Δ*j* = (*j*_charge_−*j*_off charge_) at –0.5 V vs Ag/AgCl against the scan rate. The liner slop is twice of the double-layer capacitance *C*_dl_. The O_2_ measurements were obtained by gas chromatography.

### Data availability

The data that support the findings of this study are available within the article (and its Supplementary Information files) and from the corresponding authors upon reasonable request.

## Electronic supplementary material

Supplementary Information
