# Supplementary material for: Dendritic core-shell nickel-iron-copper metal/metal oxide electrode for efficient electrocatalytic water oxidation
Source: Nat Commun. 2018 Jan 26;9:381. doi: 10.1038/s41467-017-02429-9 (PMC5786058; doi:10.1038/s41467-017-02429-9)
Supplement: Supplementary file 1 — Supplementary Information [file 41467_2017_2429_MOESM1_ESM.pdf]

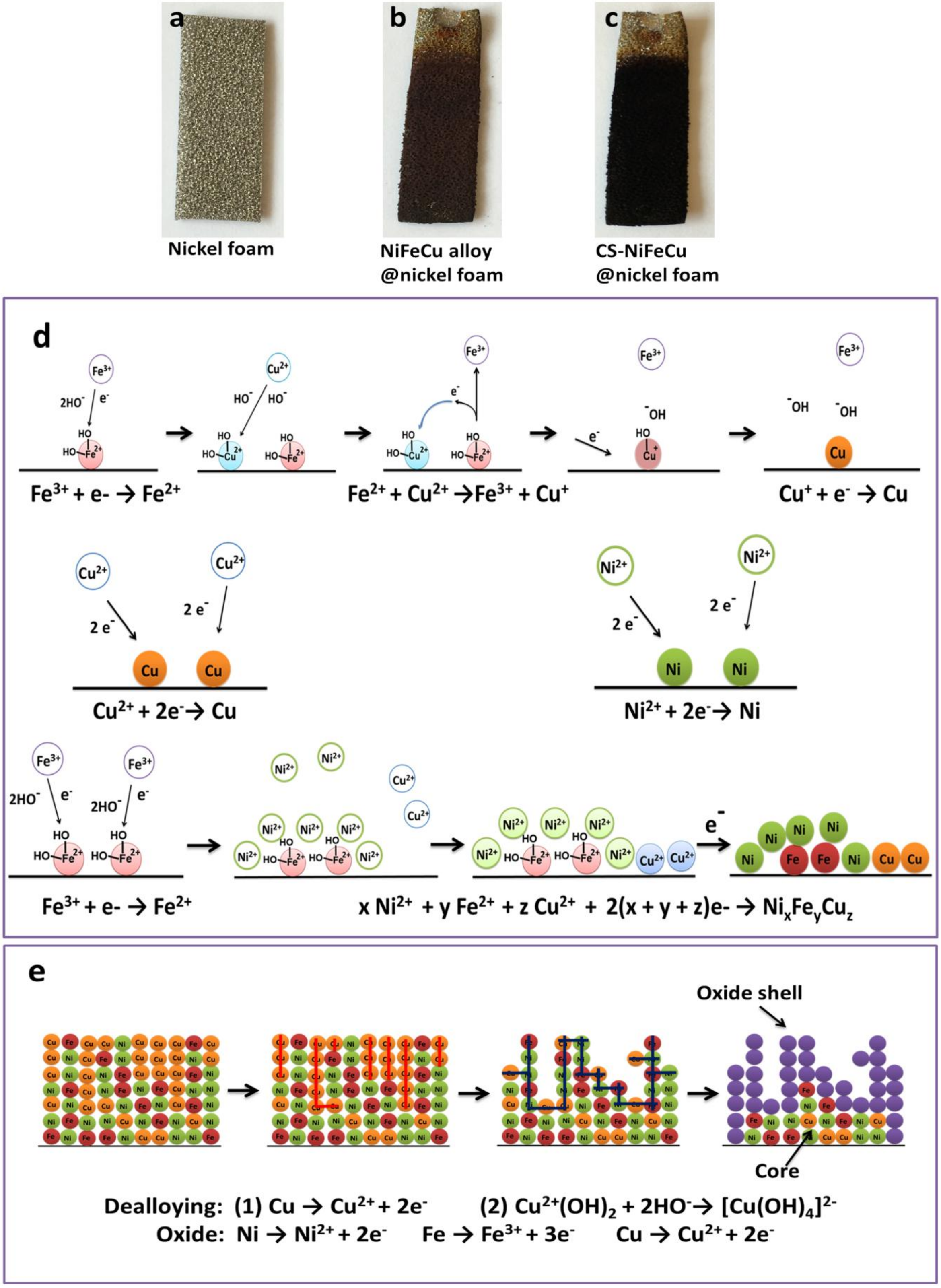

Supplementary Figure 1. The images and simplified mechanism of the preparation process. The images of **a** Ni foam, **b** the as-prepared NiFeCu alloy and **c** the CS-NiFeCu electrode. **d** A simplified electrodeposition mechanism of the as-prepared NiFeCu alloy. **e** A simplified dealloying mechanism of the NiFeCu alloy to prepare CS-NiFeCu electrode.

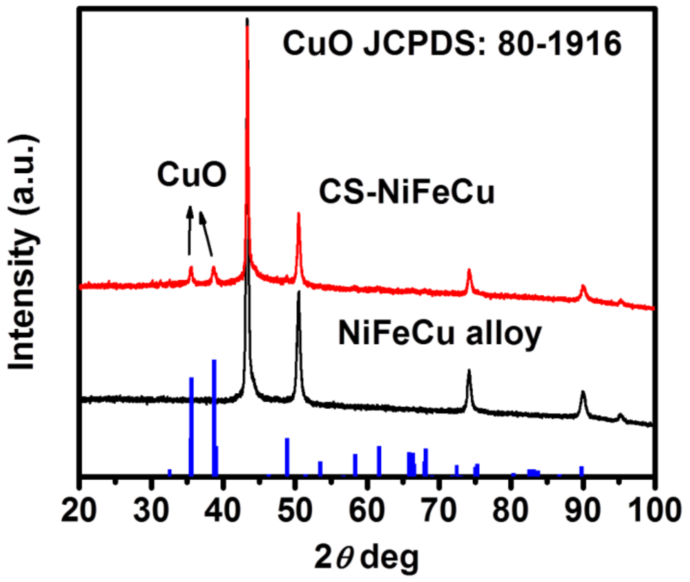

Supplementary Figure 2. The XRD patterns of the as-prepared NiFeCu alloy and CS-NiFeCu.

(Black: the as-prepared NiFeCu alloy. Red: CS-NiFeCu. Blue: The fixed-slit intensities of CuO)

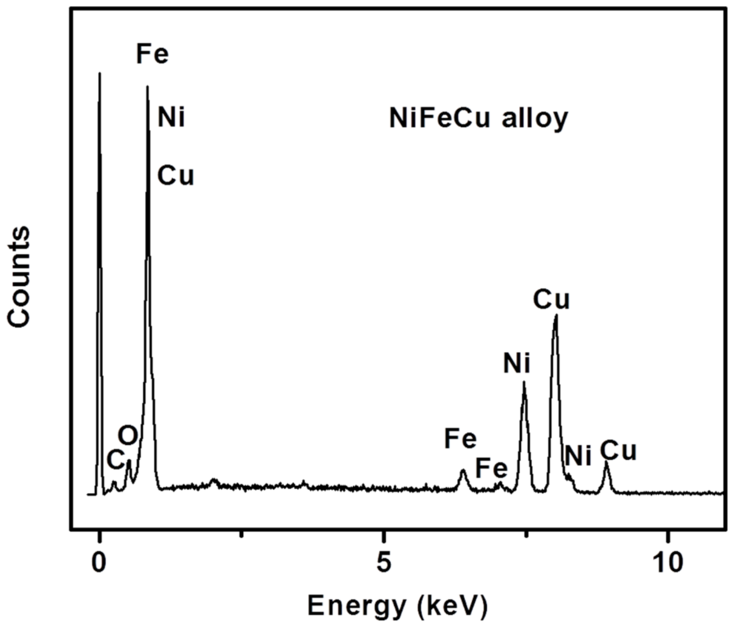

Supplementary Figure 3. The EDS pattern of NiFeCu parent alloy.

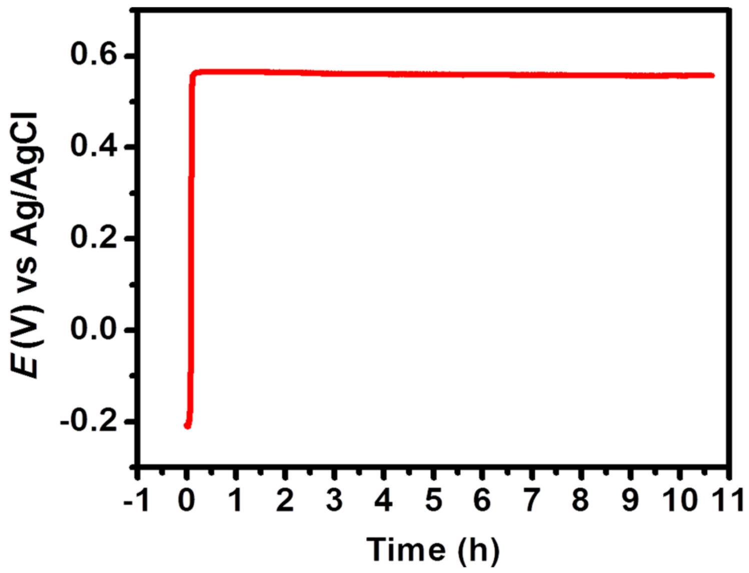

Supplementary Figure 4. The controlled current density dealloying process of NiFeCu parent alloy to CS-NiFeCu anode.

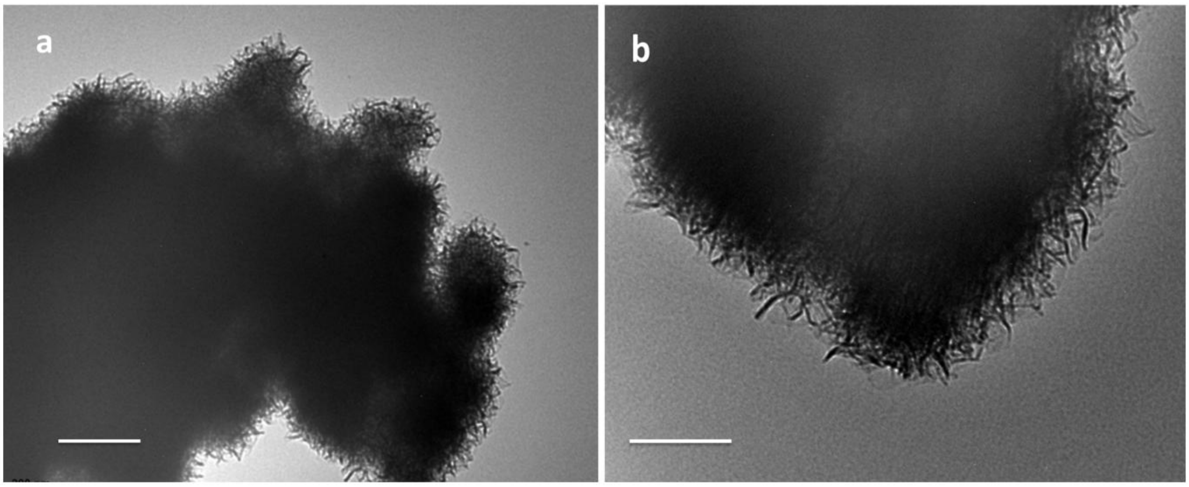

Supplementary Figure 5. TEM images of the as-prepared CS-NiFeCu branch tips. Scale bar in **a** is 200 nm, in **b** is 100 nm.

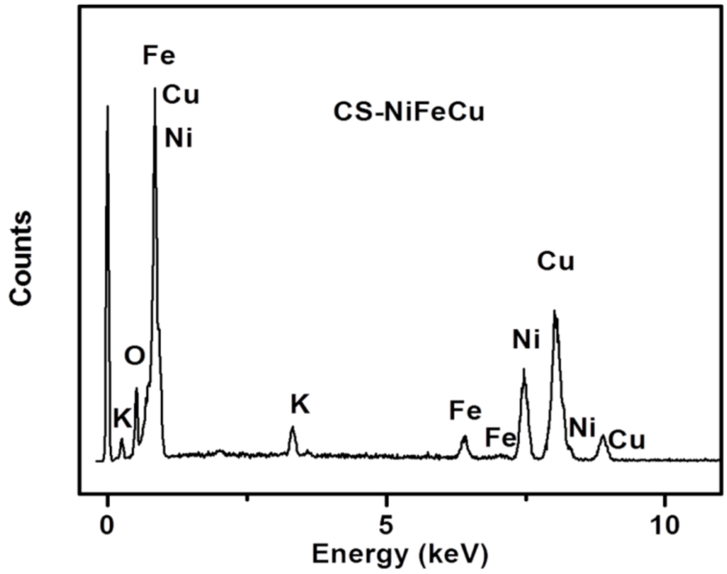

Supplementary Figure 6. The EDS pattern of CS-NiFeCu (bulk).

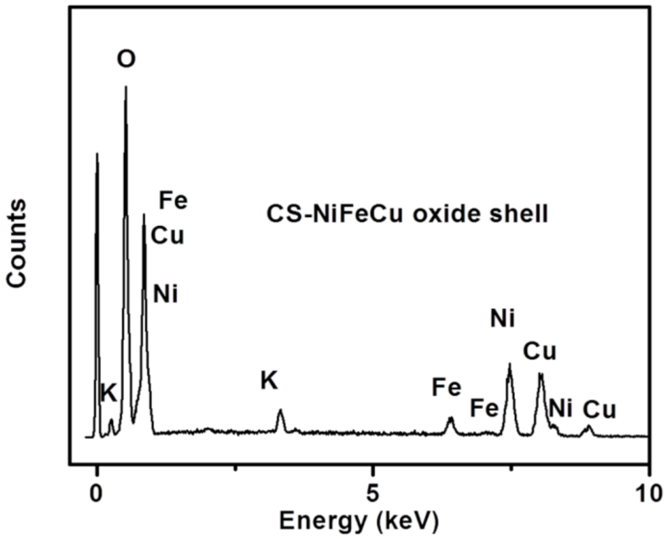

Supplementary Figure 7. The EDS pattern of CS-NiFeCu oxide shell.

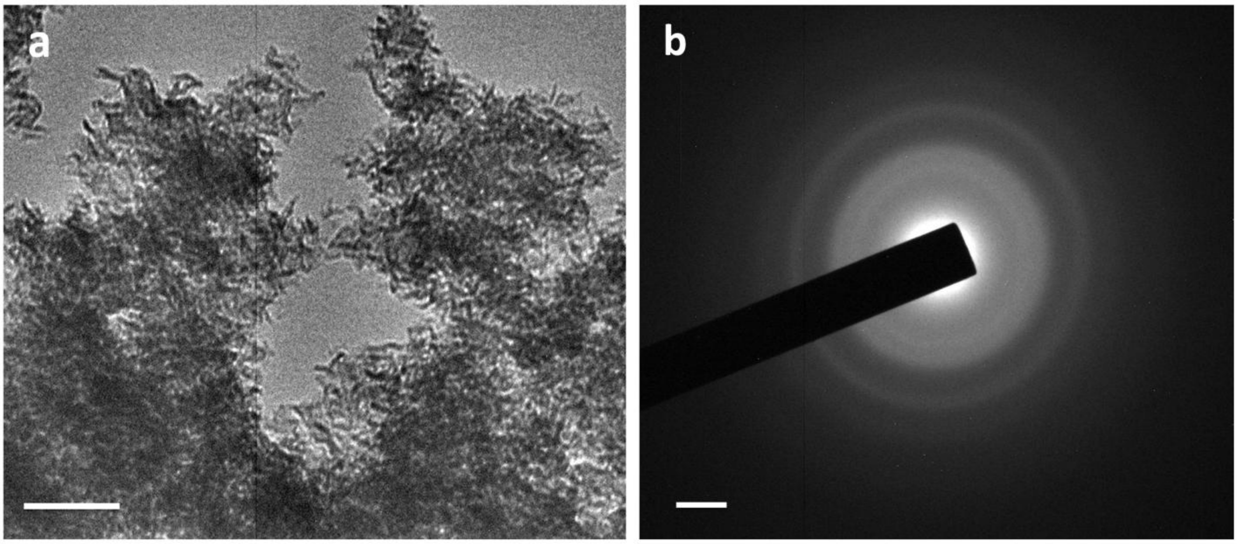

Supplementary Figure 8. The selected area electron diffraction (SAED) pattern of the oxide shell. a The image of selected oxide shell and **b** corresponding SAED. Scale bar in **a** is 50 nm, in **b** is 2 1/nm. The SAED pattern of the oxide shell is typically a halo feature, indicated that the oxide shell is in an amorphous state.

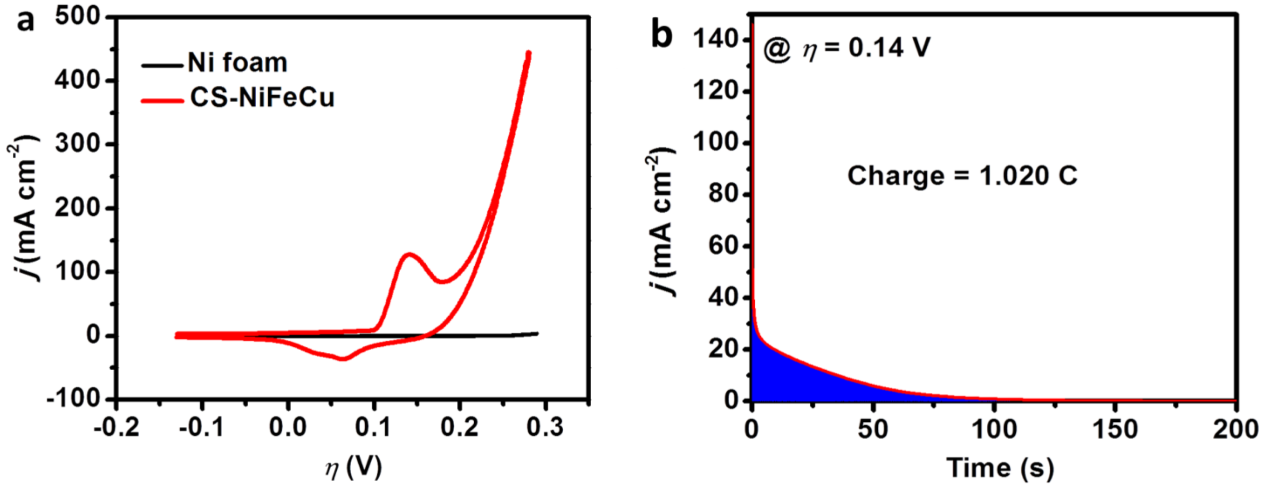

Supplementary Figure 9. The CV and controlled potential electrolysis of CS-NiFeCu. a The CV of CS-NiFeCu in 1M KOH. **b** The controlled potential electrolysis of CS-NiFeCu in 1M KOH at an overpotential of 0.14 V.

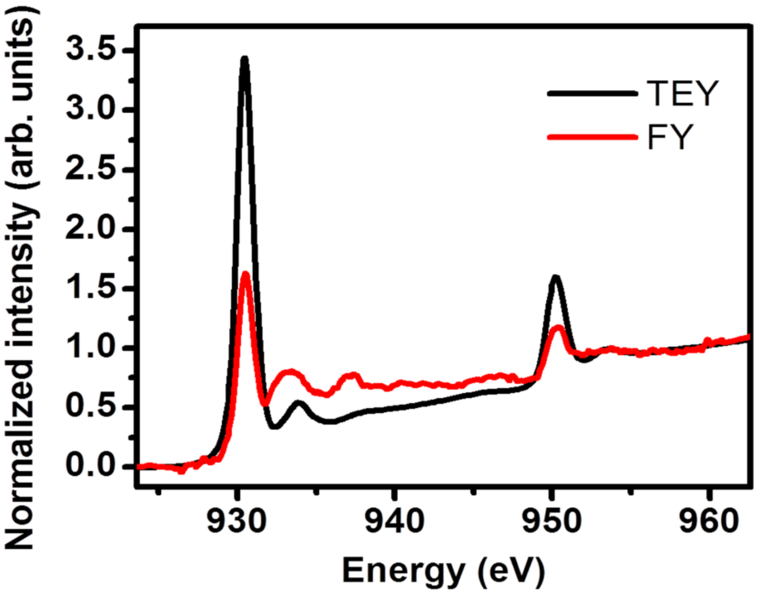

Supplementary Figure 10. Normalized soft-XAS Cu $L_{2,3}$-edge spectra of CS-NiFeCu collected in total electron yield (TEY) and fluorescence yield (FY) measurements.

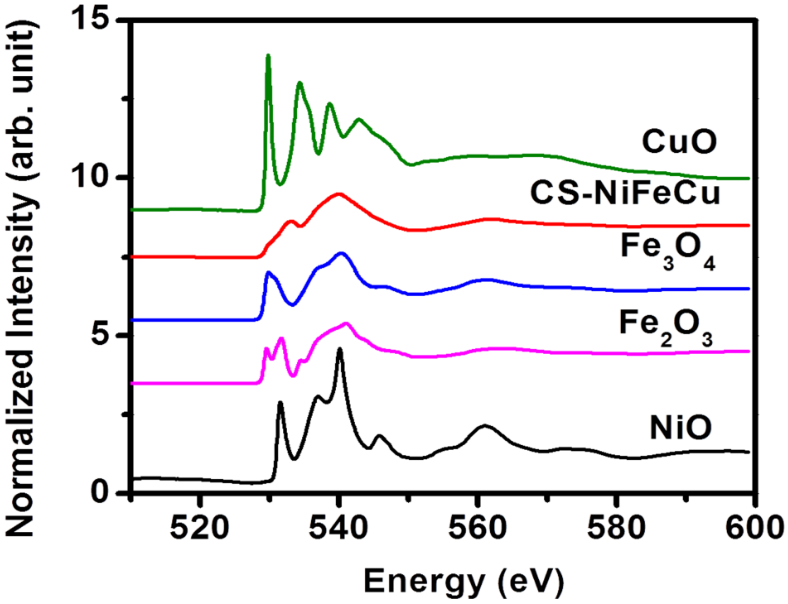

Supplementary Figure 11. Normalized soft-XAS O *k*-edge spectra of CS-NiFeCu together with NiO, Fe_3O_4, Fe_2O_3 and CuO as references.

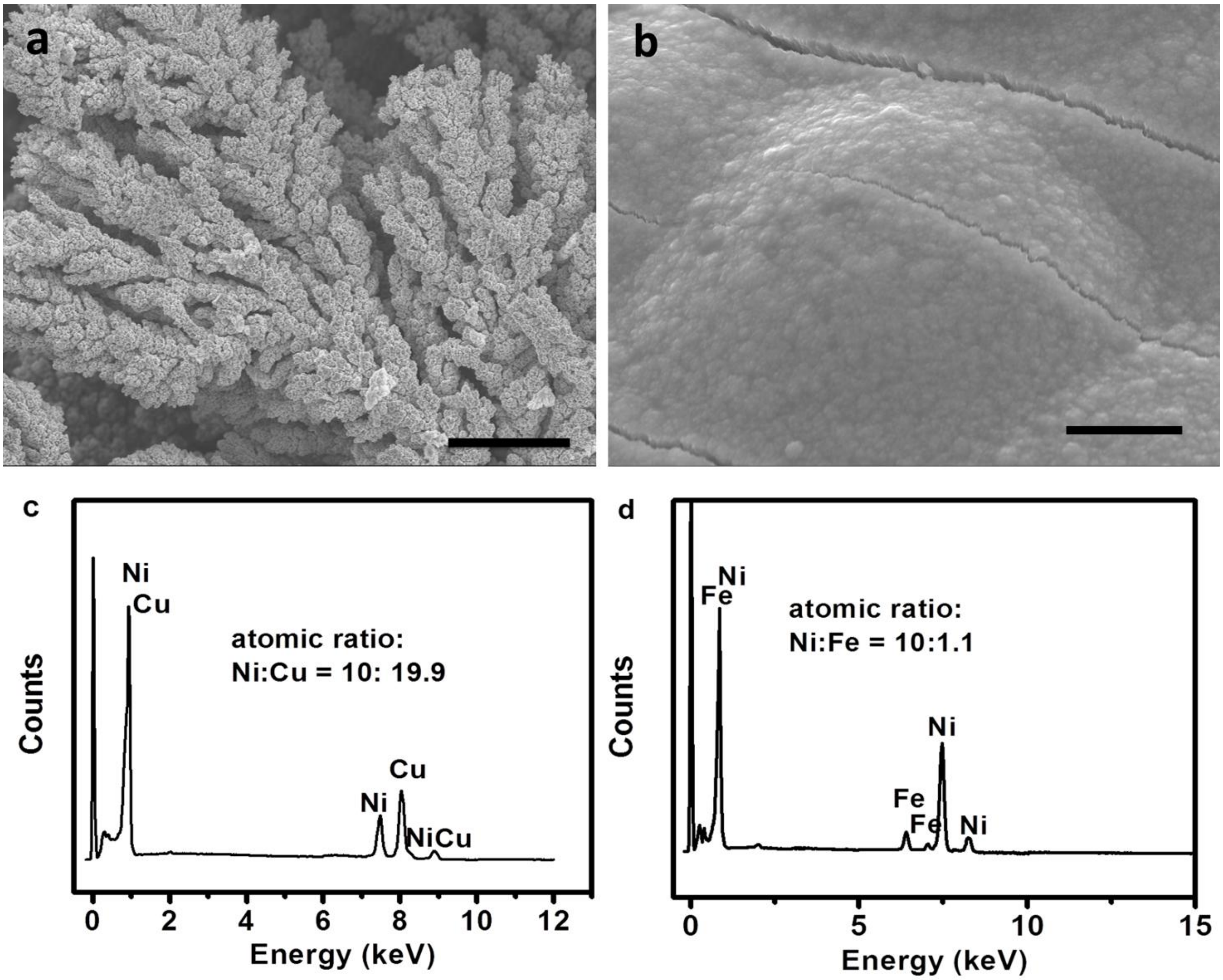

Supplementary Figure 12. SEM images and EDS spectra of NiCu and NiFe. SEM images of **a** NiCu and **b** NiFe. EDS spectra of **c** NiCu and **d** NiFe. Scale bars in **a** and **b** are 2 μm**.** The electrodeposition of NiCu was conducted at −1.1 V vs. Ag/AgCl in citrate solutions containing $Ni(Cl)_2$ $6H_2O$ (80 mM) and $Cu(SO_4)$ $5H_2O$ (40 mM). The electrodeposition of NiFe was conducted at −1.1 V vs. Ag/AgCl in citrate solutions containing $Ni(Cl)_2$ $6H_2O$ (80 mM) and $Fe(NO_3)_3$ ·$10H_2O$ (25 mM).

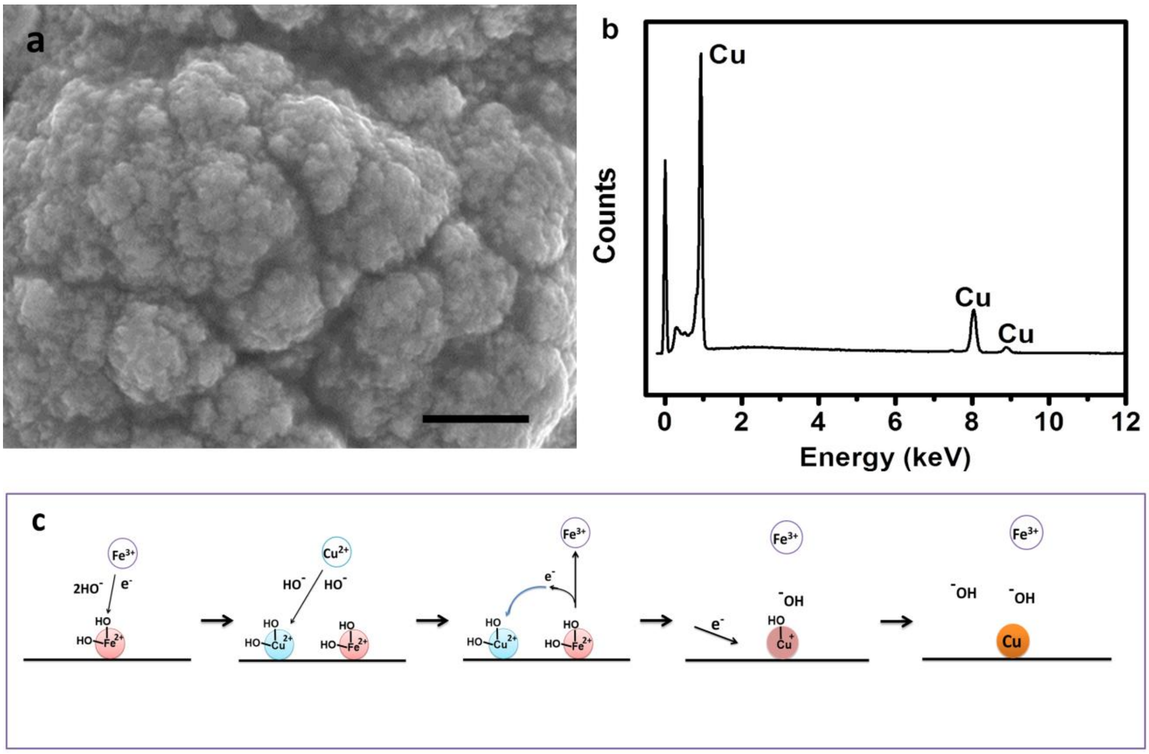

Supplementary Figure 13. The SEM image and EDS spectra of FeCu which was electrodeposited under the same conditions as that of CS-NiFeCu. a SEM image and **b** EDS spectra of the electrode after electrodeposition. Scale bar in **a** is 2 μm The electrodeposition of FeCu under the same conditions as that of CS-NiFeCu was not successful. The electrodeposition was performed at −1.1 V vs. Ag/AgCl in citrate solutions containing $Fe(NO_3)_3 \cdot 10H_2O$ (25 mM) and $Cu(SO_4) \cdot 5H_2O$ (40 mM), which leads to the formation of a Cu film, as shown above. **c** A simplified electrodeposition mechanism to explain why CuFe could not be prepared by electrodeposition. Conditions: Controlled potential, −1.1 V vs. Ag/AgCl; pH, 10.

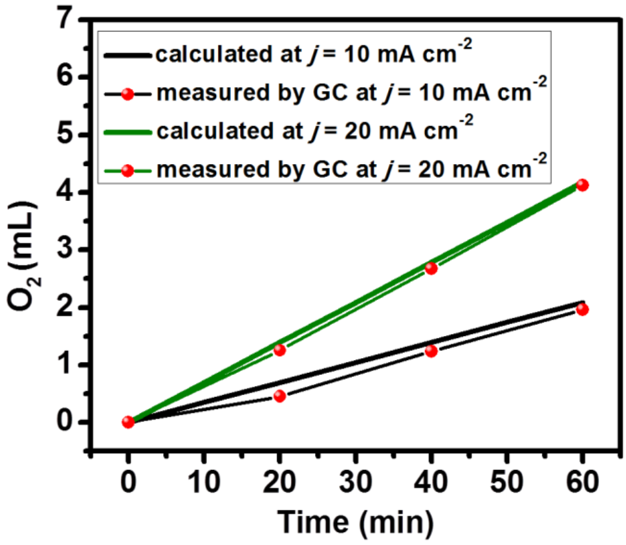

Supplementary Figure 14. The Faradaic efficiency of CS-NiFeCu for OER in 1 M KOH. The Faradaic efficiency was determined by comparing the measured amount of oxygen gas and the theoretical value calculated on the basis of the transferred charge. The quantitative yields of 93% and 98% were obtained at j = 10 and 20 mA cm^{-2}, respectively.

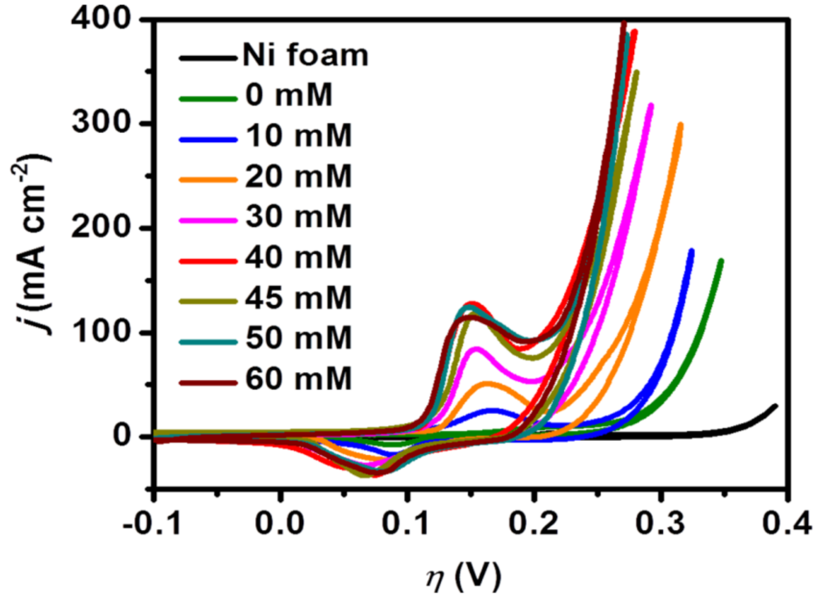

Supplementary Figure 15. Cyclic voltammograms (CVs) of CS-NiFeCu$_x$ electrodes. CS-NiFeCu$_x$ electrodes are prepared in the same method as CS-NiFeCu with constant concentration of Ni^{2+} (80 mM) and Fe^{3+} (25 mM), and various concentrations of Cu^{2+} (0–60 mM) in the electrodeposited electrolytes.

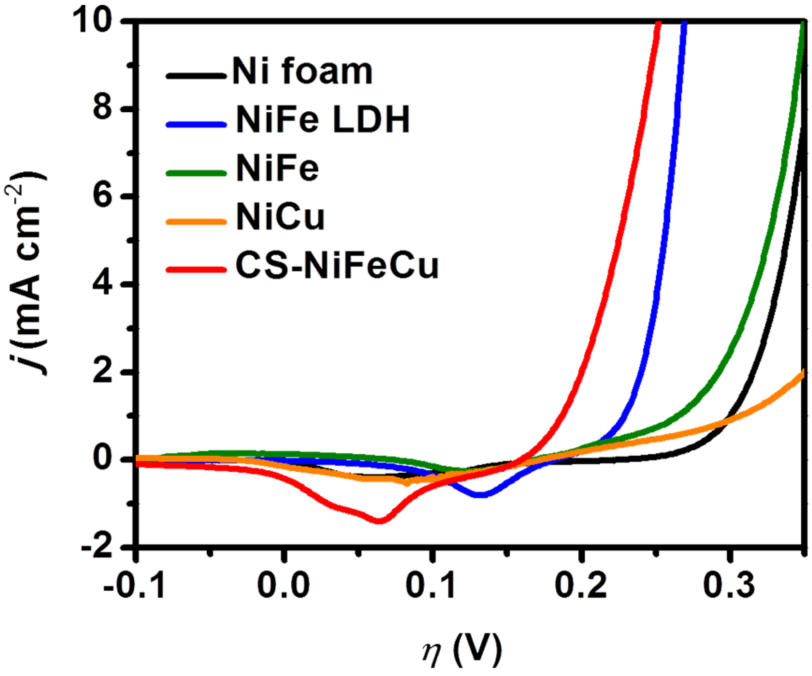

Supplementary Figure 16. Normalized LSVs of CS-NiFeCu, NiFe LDH, NiFe, and NiCu electrodes in 1 M KOH. The Ni foam with 1 cm^2 geometry surface area was used as reference with a double layer capacitance (C_{dl}) of 1.82 mF cm^{-2}. The electrochemical active surface areas (ECSA) of each working electrodes was calculated as the following equation: ECSA = C_{dl}/1.82 mF cm^{-2}. The electrochemical data (LSVs) of CS-NiFeCu, NiFe LDH, NiFe, and NiCu are normalized to ECSA, respectively, as shown in Supplementary Figure 16.

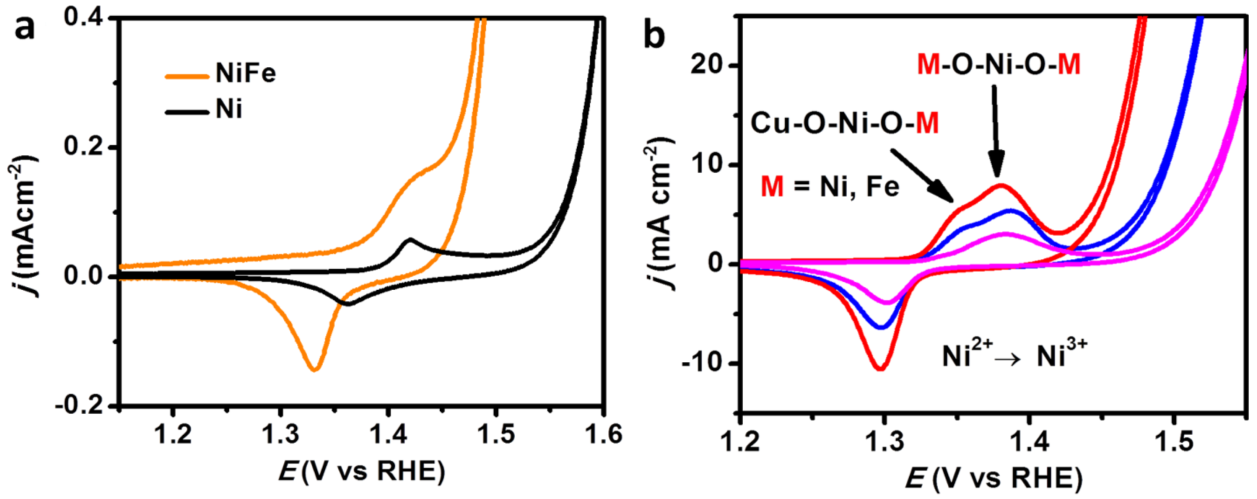

Supplementary Figure 17. Cyclic voltammograms of a Ni, NiFe and b CS-NiFeCux with nickel microelectrode as the substrate. CS-NiFeCux electrodes are prepared in the same method as CS-NiFeCu with constant concentration of Ni^{2+} (80 mM) and Fe^{3+} (25 mM), and various concentrations of Cu^{2+} [20 mM (purple), 30 mM (blue) and 40 mM (red)] in the electrodeposited electrolytes.

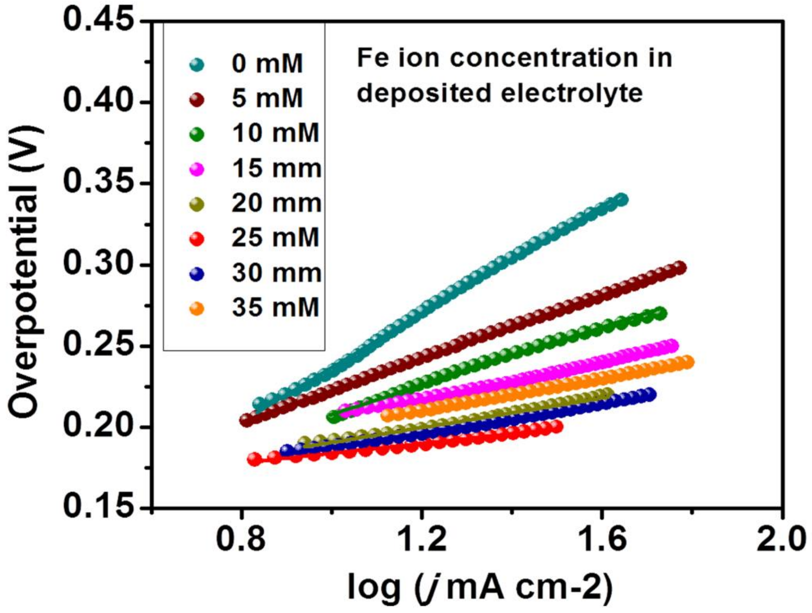

Supplementary Figure 18. Tafel slopes of CS-NiFe$_x$Cu electrodes. The electrodeposition of CS-NiFe$_x$Cu was performed under the same conditions as that of CS-NiFeCu. The electrodeposition was conducted at −1.1 V vs. Ag/AgCl in citrate solutions containing $Ni(Cl)_2 \cdot 6H_2O$, $Cu(SO_4) \cdot 5H_2O$ and $Fe(NO_3)_3 \cdot 10H_2O$. CS-NiFe$_x$Cu electrodes are prepared in the electrodeposited electrolytes with constant concentration of Ni^{2+} (80 mM), Cu^{2+} (40 mM) and variable concentrations of Fe^{3+} (0−35 mM).

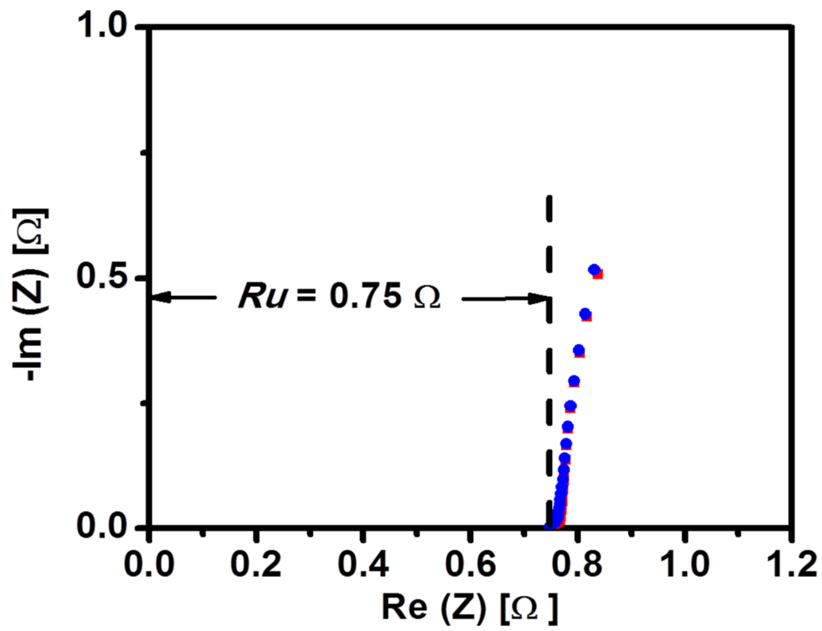

Supplementary Figure 19. Determination of the resistance *R*u for the CS-NiFeCu in 1 M KOH from a Nyquist plot. Re (Z): the real part of impedance, -Im (Z): imaginary part of impedance, *R*u: uncompensated resistance.

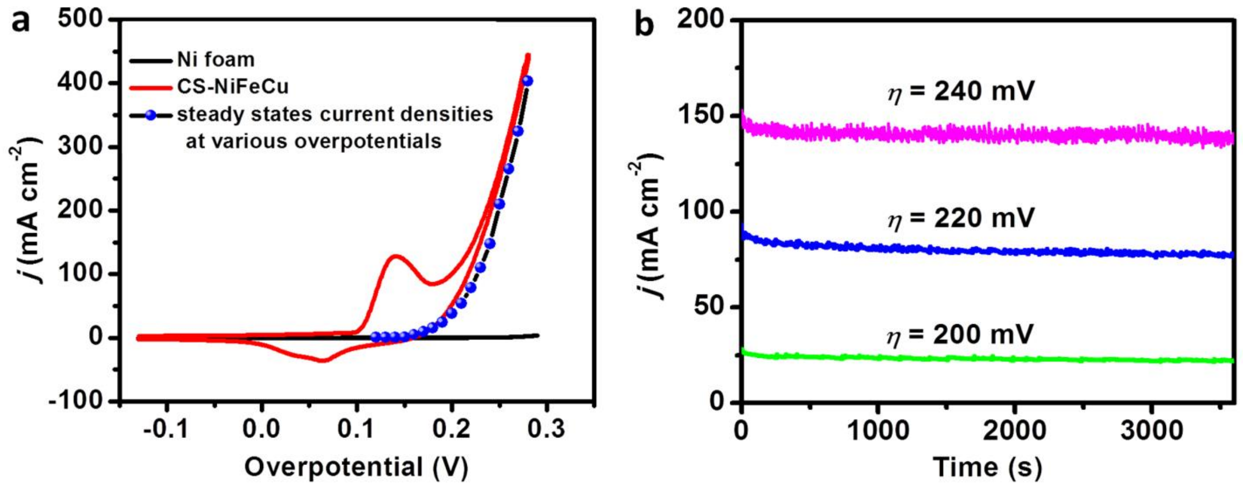

Supplementary Figure 20. The CV and steady-state electrochemical properties of CS-NiFeCu in 1.0 M KOH. a The CV of CS-NiFeCu electrode in 1.0 M KOH and the polarization curve of CS-NiFeCu electrode based on the steady-state current densities at various overpotentials. **b** The steady-state current densities of CS-NiFeCu electrode at various overpotentials in 1.0 M KOH.

Supplementary Table 1. Comparison of catalytic performance of CS-NiFeCu with reported Ni, NiFe based catalyst and IrO_2 nanoparticles.

Materials	Support	Electrolyte	Loading ($mg\ cm^{-2}$)	$\eta_{@10\ mA\ cm^{-2}}$ (mV)	Tafel slope	Ref.
CS-NiFeCu	Ni foam	1 M KOH	10.2	180	33	*This work*
IrO_2	Ni foam	1 M KOH	0.7	285	46	S1
IrO_2	carbon fiber paper	1 M KOH	3.3	264	47	S2
NiFe LDH	Ni foam	1 M KOH	8.3	244	32	S3
$Ni_xFe_{1-x}Se2$-DO	Ni foam	1 M KOH	4.1	195	28	S3
Ni_3Se_2	Cu foam	1 M KOH	3	284	80	S4
NiSe	Ni foam	1 M KOH	2.8	251	64	S5
NiFe LDH/r-GO	Ni foam	1 M KOH	0.25	195	39	S6
NiFe LDH/r-GO	Ni foam	1 M KOH	0.25/1	200/210	40	S7
EG/$Co_{0.85}Se$/NiFe LDH	Griphite foil	1 M KOH	4	203	57	S8
Ni_2P	glassy carbon	1 M KOH	0.14	290	47	S9
NiV LDH	glassy carbon	1 M KOH	0.143	270	50	S10
$Ni_{60}Fe_{30}Mn_{10}$		0.5 M KOH		200	62	S11
Gelled FeCoW oxy-hydroxide	Gold foam	1 M KOH		191		S12
$Co_{0.37}Ni_{0.26}Fe_{0.37}O$	3D carbon fiber	1 M KOH		232	37.6	S13
NiFe hydroxide/graphene superlattice	glassy carbon	1 M KOH	0.25	210	40	S7

Supplementary Notes

Supplementary Note 1. Electrodeposition mechanism of NiCuFe alloy. (Supplementary Fig. 1d)

Electrodeposition pathways of Ni, Fe, and Cu.

Ⅰ. Cu was electrodeposited through two pathways:

1) electro-reduction pathway: $Cu^{2+} + 2e^{-} \rightarrow Cu$;

2) electrochemical reduction pathway: Fe^{3+} + e- → Fe^{2+}, $Fe^{2+} + Cu^{2+} \rightarrow Fe^{3+} + Cu^{+}$, $Cu^{+} + e^{-} \rightarrow Cu$.

Ⅱ. Ni was electrodeposited through electro-reduction pathway: $Ni^{2+} + 2e^{-} \rightarrow Ni$

Ⅲ. Fe was electrodeposited with Ni through electro-reduction pathway under the protection of Ni iron ($Fe^{2+} + Ni^{2+}$ + 4e- → Fe + Ni).

Under the electrodeposited conditions, Fe^{3+} shows good solubility. The electrodeposition of Fe occurs when ferrous hydroxide intermedia species adhered on the substrate[14,15]. Firstly, Fe^{3+} is reduced to Fe^{2+}, and the pH value is high enough to cause the formation of ferrous hydroxide, which adsorbs preferentially on the electrode. Secondly, the Ni ions show competitive behavior with Cu^{2+}. Ferrous hydroxide can be further reduced to generate NiFe alloy when Ni ions protect the ferrous hydroxide from Cu^{2+} ion. Otherwise, ferrous hydroxide will be oxidized by Cu^{2+} to generate Fe^{3+} and Cu^{+}. Cu^{+} got one more electron from the cathode and been reduced to Cu metal atom ($Cu^{+} + e^{-} \rightarrow Cu$).

During the electrodeposition process, Ni, Fe, and Cu metal atoms are deposited on the substrate at the same time without order.

Supplementary Note 2. A simplified dealloying mechanism of NiCuFe alloy to synthesis CS-NiFeCu.

(Supplementary Fig. 1e)

Ⅰ. Under the dealloying conditions, copper shows the highest chemical activity relative to nickel and iron. Therefore, during the process, loaded Cu on the surface was gradually oxidized. Copper prevails at the convex parts was oxidized and dissolved into the solution. Copper doped in NiFe atomic layer was oxidized to generate the NiFeCu oxide component.

$Cu \rightarrow Cu^{2+} + 2e^{-}$

$Cu^{2+}(OH)_2 + 2HO^{-} \rightarrow [Cu(OH)_4]^{2-}$

Ⅱ. During dealloying, a metal oxide layer, including NiFe oxide and NiFeCu oxide, was generated on the surface and prevented the metallic core from further corrosion.

$Ni \rightarrow Ni^{2+} + 2e^{-}$, $Fe \rightarrow Fe^{3+} + 3e^{-}$, $Cu \rightarrow Cu^{2+} + 2e^{-}$

Metal oxide: NiFe oxide and NiFeCu oxide

Supplementary Note 3. How to calculate the amount of involved catalyst for OER? (Supplementary Fig. 9)

The process for the conversion of the Ni^{2+} to Ni^{3+} species is not a pure electron losing reaction.[16] Here, we presume it a one-electron process. The amount of involved Ni for OER was calculated from the charge built up of Ni^{2+} to Ni^{3+} controlled potential electrolysis at 0.14 V.[16] A total charge of 1.020 C was obtained, indicated that ca. 0.599 mg cm^{-2} Ni^{2+} was involved, corresponding a loading amount of ca. 1.6 mg cm^{-2} calculated based on the atomic proportion of Ni:Fe:Cu:O =10:1:10:26.

Supplementary Note 4. The electrodeposition of FeCu under the same conditions as that of CS-NiFeCu was not successful. Why? (Supplementary Fig. 13)

The electrodeposition of Fe occurs when ferrous hydroxide intermedia species adhered on the substrate[s15,s16]. Under the electrodeposition conditions, Fe^{3+} was reduced to Fe^{2+}, and the pH value is high enough to cause the formation of ferrous hydroxide, which adsorbs preferentially on the electrode ($Fe^{3+} + e^{-} \rightarrow Fe^{2+}$). Under the electrodeposited conditions, Fe^{3+} shows good solubility. Cu^{2+} complex shows high oxidizability, which could oxidize Fe^{2+} to generate Fe^{3+} and Cu^{+} ($Fe^{2+} + Cu^{2+} \rightarrow Fe^{3+} + Cu^{+}$). Cu^{+} got one more electron from the cathode and been reduced to Cu metal atom ($Cu^{+} + e^{-} \rightarrow Cu$). This reaction prevents Fe ions from electrodeposition.

Supplementary Note 5. Why we recorded the polarization curves from high initial potentials to low final potentials?

The polarization curves were recorded from high initial potentials to low final potentials with a quiescent time of 5 s at initial potentials. The CV of CS-NiFeCu in 1M KOH was recorded as shown in Supplementary Fig. 20. A broad oxidation peak was observed from 0.1 V to 0.2 V, when the

potential scan from −0.1 V to 0.3 V, following with a current density for OER. A typical overlap between Ni^{2+}/Ni^{3+} oxidation and OER was observed as shown in Supplementary Fig. 20. Furthermore, a polarization curve without current density overlap was obtained when the potential scan from 0.3 V to −0.1 V. The polarization curve from 0.3 V to −0.1 V shows similar activity compared with the steady-state current densities at various overpotentials. These results indicate that, in our case, the polarization curve from 0.3 V initial potential to −0.1 V final potential could provide more reliable information compared to the scan from low initial potential to high final potential.

Supplementary Methods

Materials. The starting materials of nickel(II) chloride hexahydrate ($NiSO_4 \cdot 6H_2O$), iron(III) nitrate nonahydrate ($Fe(NO_3)_2 \cdot 9H_2O$), copper(II) sulfate pentahydrate ($CuSO_4 \cdot 5H_2O$), NaOH, Sodium citrate tribasic dehydrate ($Na_3C_6H_5O_7 \cdot 2H_2O$), ammonium sulfate ($(NH_4)_2SO_4$) and sodium hypophosphite monohydrate ($NaPO_2H_2 \cdot H_2O$) were purchased from Sigma-Aldrich and used as received. The water used for electrolyte solutions was deionized with a Millipore Milli-Q UF Plus system (15–18 MΩ-cm resistivity). Nickel foam with a thickness of 1.6 mm was purchased from Sigma-Aldrich. The foam was cut into 1×2 cm^2 pieces and used as electro-deposition substrates.

Supplementary References

1. Yan, X. *et al*. From Water oxidation to reduction: transformation from $Ni_xCo_{3-x}O_4$ nanowires to NiCo/NiCoOx heterostructures. *ACS Appl. Mater. Interfaces* **8,** 3208–3214 (2016).
2. Wang, P., Song, F., Amal, R., Ng, Y. H. & Hu, X. Efficient water splitting catalyzed by cobalt phosphide-based nanoneedle arrays supported on carbon cloth. *ChemSusChem* **9,** 472–477 (2016).
3. X. Xu, F. Song, X. Hu, A nickel iron diselenide-derived efficient oxygen-evolution catalyst. *Nature Comm.* **7,** 12324 (2016).
4. Shi, J. L., Hu, J. M., Luo, Y. L., Sun, X. & Asiri, A. M. Ni_3Se_2 film as a non-precious metal bifunctional electrocatalyst for efficient water splitting. *Catal Sci Technol* **5,** 4954–4958 (2015).
5. Tang, C., Cheng, N., Pu, Z., Xing, W. & Sun, X. NiSe Nanowire Film Supported on Nickel Foam: An Efficient and Stable 3D Bifunctional Electrode for Full Water Splitting. *Angew. Chem. Int. Ed.* **54,**9351–9355 (2015).
6. Long, X. *et al*. A strongly coupled graphene and FeNi double hydroxide hybrid as an excellent electrocatalyst for the oxygen evolution reaction. *Angew. Chem. Int. Ed.* **53,** 7584–7588 (2014).
7. Ma, W. *et al*. A Superlattice of Alternately Stacked Ni–Fe Hydroxide Nanosheets and Graphene for Efficient Splitting of Water. *ACS Nano* **9,** 1977–1984 (2015).
8. Hou, Y. *et al.* Vertically oriented cobalt selenide/NiFe layered-double-hydroxide nanosheets supported on exfoliated graphene foil: an efficient 3D electrode for overall water splitting. *Energy Environ Sci* **9,** 478–483 (2016).
9. Stern, L. A., Feng, L. G., Song, F. & Hu, X. L. Ni_2P as a Janus catalyst for water splitting: the oxygen evolution activity of Ni_2P nanoparticles. *Energy Environ. Sci.* **8,** 2347–2351 (2015).
10. Fan, K. *et al.* Nickel–vanadium monolayer double hydroxide for efficient electrochemical water oxidation. *Nature Comm.* **7,** 11981 (2016).
11. Detsi, E. *et al.* Mesoporous $Ni_{60}Fe_{30}Mn_{10}$-alloy based metal/metal oxide composite thick films as highly active and robust oxygen evolution catalysts. *Energy Environ. Sci.* **9,** 540–549 (2016).
12. Zhang, B. *et al*. Homogeneously dispersed multimetal oxygen-evolving catalysts. *Science* **352,**

333–337 (2016).

13. Chen, W. *et al.* In Situ Electrochemical Oxidation Tuning of Transition Metal Disulfides to Oxides for Enhanced Water Oxidation. *ACS Central Sci.* **1,** 244–251 (2015).
14. Dahms, H. The influence of hydrolysis on the deposition and CO-DE-position of iron-group metals (Fe, Co, Ni) at the dropping mercury electrode. *J. Electroanal. Chem.* **8**, 5–12 (1964).
15. Dahms, H. Dahms & Croll, I. M. The anomalous codeposition of iron-nickel alloys. *J. Electrochem. Soc.* **112**, 771–775 (1965)
16. Louie, M. W. & Bell, A. T. An investigation of thin-film Ni–Fe oxide catalysts for the electrochemical evolution of oxygen. *J. Am. Chem. Soc.* **135**, 12329–12337 (2013).
